# Combined Analysis of Transcriptome and Metabolome Explains the Differences in Pulp Color in Jackfruit

**DOI:** 10.1002/pld3.70127

**Published:** 2026-02-11

**Authors:** Jianjun Liang, Xiangwei Ma, Chenxin Yi, Hailan Zhou, Zhuangmin Wei, Xiuguan Tang, Weiyan Ye, Hailing Tang, Pengjin Zhu

**Affiliations:** ^1^ Guangxi Subtropical Crops Research Institute Guangxi Academy of Agricultural Sciences Nanning China; ^2^ Guangxi Key Laboratory of Quality and Safety Control for Subtropical Fruits Nanning China; ^3^ Key Laboratory of Quality and Safety Control for Subtropical Fruit and Vegetable Ministry of Agriculture and Rural Affairs Nanning China; ^4^ School of Tropical Agriculture and Forestry Hainan University Haikou China

**Keywords:** carotenoid biosynthesis, flavonoid biosynthesis, fruit pigmentation, jackfruit, metabolome, pulp color, transcriptome

## Abstract

The pulp color of jackfruit reflects variations in its nutritional composition and influences market preference. We investigated the mechanism underlying pulp coloration through an integrated transcriptomic and metabolomic analysis of three jackfruit cultivars—light yellow “THA”, yellow “GTM”, and orange “YNH”. Twenty‐five differentially accumulated flavonoids and 32 differentially accumulated carotenoids were identified. Naringenin chalcone, eriodictyol, taxifolin, zeaxanthin, and lutein were identified as the key flavonoids and carotenoids associated with the light‐yellow tone of THA pulp. Phlorizin and lutein were associated with the yellow tone of GTM pulp, whereas apigenin, luteolin, zeaxanthin, and violaxanthin dipalmitate were the key pigments regulating the orange tone of YNH pulp. Differentially expressed genes involved in flavonoid and carotenoid biosynthesis included *PAL*, *C4H*, *4CL*, *F3H*, *FLS*, *ANS*, *ANR*, *PSY*, *PDS*, *ZDS*, *LCYE*, *LUT5*, *ZEP*, and *VDE*. This study provides a foundation for elucidating the molecular mechanisms underlying jackfruit pulp coloration at transcriptional and metabolic levels.

## 1 Introduction

Jackfruit ( *Artocarpus heterophyllus* Lam.) is one of the most important tropical fruits in the Moraceae family (Prakash et al. 2009; Zerega et al. 2010). The edible parts of ripe jackfruit include the pulp and seeds. Pulp color is a key trait influencing nutritional composition, flavor, taste, consumer preference, and market value (Kavya et al. 2019; Khan et al. 2021; Natta et al. 2023). Depending on the variety, jackfruit pulp color ranges from white to light yellow, yellow, deep yellow, lemon yellow, light saffron, saffron, deep saffron, or orange (Jagadeesh et al. 2007; Ranasinghe et al. 2019). Jackfruit pulp is rich in nutritional ingredients such as carbohydrates, proteins, lipids, vitamins, and minerals, as well as phytochemicals such as polyphenols, carotenoids, and flavonoids (Baliga et al. 2011; Swami et al. 2012).

The fruit color is primarily determined by specific pigments, including flavonoids, anthocyanins, and carotenoids, as well as the expression of related biosynthetic genes (Tanaka et al. 2008; Tang et al. 2024). Flavonoids constitute a large class of polyphenolic secondary metabolites and are divided into several groups, including chalcones, flavonols, flavones, flavanones, flavanols, isoflavones, and anthocyanins (Zhang et al. 2021). Flavonoids play diverse and vital roles in plants, acting as signaling molecules, phytoalexins, detoxifying agents, and regulators of seed germination and stress tolerance (Samanta et al. 2011; Baskar et al. 2018; Shomali et al. 2022). Flavonoids, particularly flavonols, flavones, chalcones, and anthocyanins, are key contributors to plant pigmentation, and flavonols, flavones, and chalcones typically impart yellow hues, whereas anthocyanins produce orange to blue hues (Tanaka et al. 2008). Phytochemical studies have identified numerous flavonoids in jackfruit, especially in the peel and pulp (Baliga et al. 2011; Zhang et al. 2017; Cheng et al. 2023; Ye et al. 2025). In addition, researchers found that 19 flavonoids accumulated predominantly in orange flesh, whereas only three flavanones accumulated in yellow flesh from the tested jackfruit samples (Cen et al. 2021).

Carotenoids, classified into carotenes and xanthophylls, are widely distributed pigments. They are responsible for the yellow to red coloration of many fruits and vegetables (Namitha and Negi 2010; Saini et al. 2015), and also play important roles in photosynthesis, photoprotection, and human health and nutrition (Domonkos et al. 2013; Hashimoto et al. 2016; Giordano and Quadro 2018; Rodriguez‐Concepcion et al. 2018). In jackfruit, the major carotenoids include all‐trans‐lutein, all‐trans‐*β*‐cryptoxanthin, all‐trans‐*α*‐carotene, all‐trans‐*β*‐carotene, *α*‐ and *β*‐zeacarotene, 5,6‐epoxy‐*β*‐carotene, crocetin, lycopene, all‐trans‐lutein, all‐trans‐neoxanthin, 9‐cis‐neoxanthin, and 9‐cis‐violaxanthin (Tee and Lim 1991; Setiawan et al. 2001; Chandrika et al. 2004; de Faria et al. 2009). Differences in carotenoid composition and concentration directly contribute to the variation in pulp colors. For example, red and orange toned pulps are enriched with *β*‐cryptoxanthine, *β*‐carotene, *α*‐carotene, lycopene and *β*‐citraurin, while yellow pulp contains violaxanthin (Hu et al. 2017; Shyamalamma et al. 2017). Despite these extensive characterization of flavonoid and carotenoid composition of jackfruits, the transcriptional and metabolic mechanisms underlying jackfruit pulp coloration remain unclear, as most of the current research mainly focuses on the analysis of the pigment components.

In this study, we performed a combined analysis of the transcriptome and metabolome of three jackfruit cultivars with different pulp colors (light yellow, yellow, and orange) to elucidate the molecular mechanisms driving pulp color formation.

## 2 Materials and Methods

### 2.1 Plant Materials

Three jackfruit cultivars—“THA” (GXRZBLM00013), “GTM” (GXRZBLM00109), and “YNH” (GXRZBLM00106)—were selected to explore the mechanism of pulp coloration. The cultivars were preserved in the Jackfruit Germplasm Resource Nursery of the Guangxi Subtropical Crops Research Institute (22.899° N, 108.343° E). Pulps from mature jackfruit (4 months after flowering) were collected for the experiments, with three biological replicates per group. And the three biological replicates of each variety were selected from three different healthy plants, with one fruit taken from each plant, and all these fruits had the same maturity. For samples used in transcriptomic analysis, the suffixes of the three biological replicates for each variety are 123 respectively. For samples used in metabolomic analysis, the suffixes of the three biological replicates for each variety are 456 respectively. All samples were frozen in liquid nitrogen and stored in a −80°C freezer for metabolomic analysis and transcriptomic sequencing, which were carried out by Wuhan Metware Biotechnology Co. Ltd. (Wuhan, China).

### 2.2 Pulp Color Index Measurement

The fruit pulp color parameters L*, a*, and b* scores were calculated using the KONICA MINOLTA CR‐10 Plus Color Reader (Japan), with a minimum of nine biological replicates. L* represents the variation in sample lightness. a* indicates the red‐green bias—higher values tend toward red, while lower values tend toward green. b* denotes the yellow‐blue bias—higher values lean toward yellow, while lower values lean toward blue.

### 2.3 Metabolomic Analysis

The extraction, detection, and quantitative analyses of metabolites in the samples were performed by Wuhan Metware Biotechnology Co. Ltd. Briefly, freshly collected jackfruit pulp samples were vacuum freeze‐dried, then weighed and extracted with a −20°C pre‐cooled 70% methanolic aqueous internal standard extract. The extracts were filtered and analyzed using ultra‐high‐performance liquid chromatography–tandem mass spectrometry (UPLC–MS/MS). All metabolites were annotated using the MetWare database and quantified using multiple reaction monitoring (MRM). Data were analyzed using Analyst 1.6.3 software (AB SCIEX, Ontario, Canada). Orthogonal partial least squares discriminant analysis (OPLS‐DA) and principal component analysis (PCA) were used to evaluate differences and reliability of metabolites in the samples. Those with variable importance in projection (VIP) ≥ 1 and |log2(fold change [FC])| ≥ 1 were identified as differentially accumulated metabolites (DAMs). Identified metabolites were annotated using the Kyoto Encyclopedia of Genes and Genomes (KEGG) compound database (http://www.kegg.jp/kegg/compound/) and mapped to the KEGG pathway database (http://www.kegg.jp/kegg/pathway.html).

### 2.4 Determination of Flavonoid Composition

Flavonoid composition was determined by Wuhan Metware Biotechnology Co. Ltd. Briefly, pulps from the three jackfruit cultivars (THA, GTM, and YNH) were collected and stored at −80 °C until use. The samples were freeze‐dried and ground into powder. Twenty milligrams of the powder was extracted with 70% methanol (0.5 mL), and 10 μL internal standard (4000 nmol/L) was added to the extract for quantification. The extract was sonicated for 30 min and then centrifuged at 12,000 g at 4 °C for 5 min. The supernatant was subsequently filtered through a 0.22 μm membrane filter for further LC–MS/MS analysis. Extracts were analyzed using a UPLC‐ESI‐MS/MS system (UPLC, ExionLC ad, https://sciex.com.cn/; MS, Applied Biosystems 6500 Triple Quadrupole, https://sciex.com.cn/). Flavonoid contents were detected by MetWare (http://www.metware.cn/) based on an AB Sciex QTRAP6500 LC–MS/MS platform.

### 2.5 Determination of Carotenoid Composition

Carotenoid composition was determined by Wuhan Metware Biotechnology Co. Ltd. Briefly, pulps from the three cultivars were collected and stored at −80 °C until use. The samples were freeze‐dried and ground into powder, and 50 mg of the powder was extracted with 0.5 mL of a mixed solution of n‐hexane, acetone, and ethanol (1:1:1, v/v/v). The extract was vortexed for 20 min at 25°C and centrifuged at 12000 r/min for 5 min at 4°C. The supernatants were collected, and the residue was re‐extracted by repeating the aforementioned steps under the same conditions. The supernatants were combined, evaporated to dryness, and reconstituted in 150 μL dichloromethane. The solution was then filtered through a 0.22 μm membrane filter for LC–MS/MS analysis. The extracts were analyzed using a UPLC‐APCI‐MS/MS system as described in section 2.4. The carotenoid contents were detected by MetWare (http://www.metware.cn/) based on an AB Sciex QTRAP 6500 LC–MS/MS platform.

### 2.6 RNA‐Seq Analysis

#### 2.6.1 Raw Data Filtration and Mapping

Data quality control was performed using fastp to remove adapter‐containing reads. The reference genome of the jackfruit cultivar “S10” (Lin et al. 2022) was downloaded from the National Center for Biotechnology Information (NCBI) Sequence Read Archive database (PRJNA788174 and PRJNA791757). HISAT was used to build and index, and clean reads were aligned to the reference genome. Gene expression levels were quantified using featureCounts to calculate gene alignment statistics. Subsequently, fragments per kilobase of transcript per million fragments mapped (FPKM) values for each gene were computed based on gene length.

#### 2.6.2 Functional Annotation and Screening of Differential Genes

Gene function annotation was performed using four primary databases: NCBI non‐redundant protein sequences (NR), Swiss‐Prot protein sequence database, KEGG, and Gene Ontology (GO). Differential expression analysis was conducted using DESeq2 software. Significantly differentially expressed genes (DEGs) were identified with a false discovery rate (FDR) < 0.05 and|log2 FC| ≥ 1.

### 2.7 Statistical Analysis

All data were analyzed using GraphPad Prism 6 and Excel 2021 and are expressed as mean ± SD Each result was obtained from three biological replicates.

## 3 Results

### 3.1 Color Phenotype of Three Jackfruit Cultivars

**FIGURE 1:**
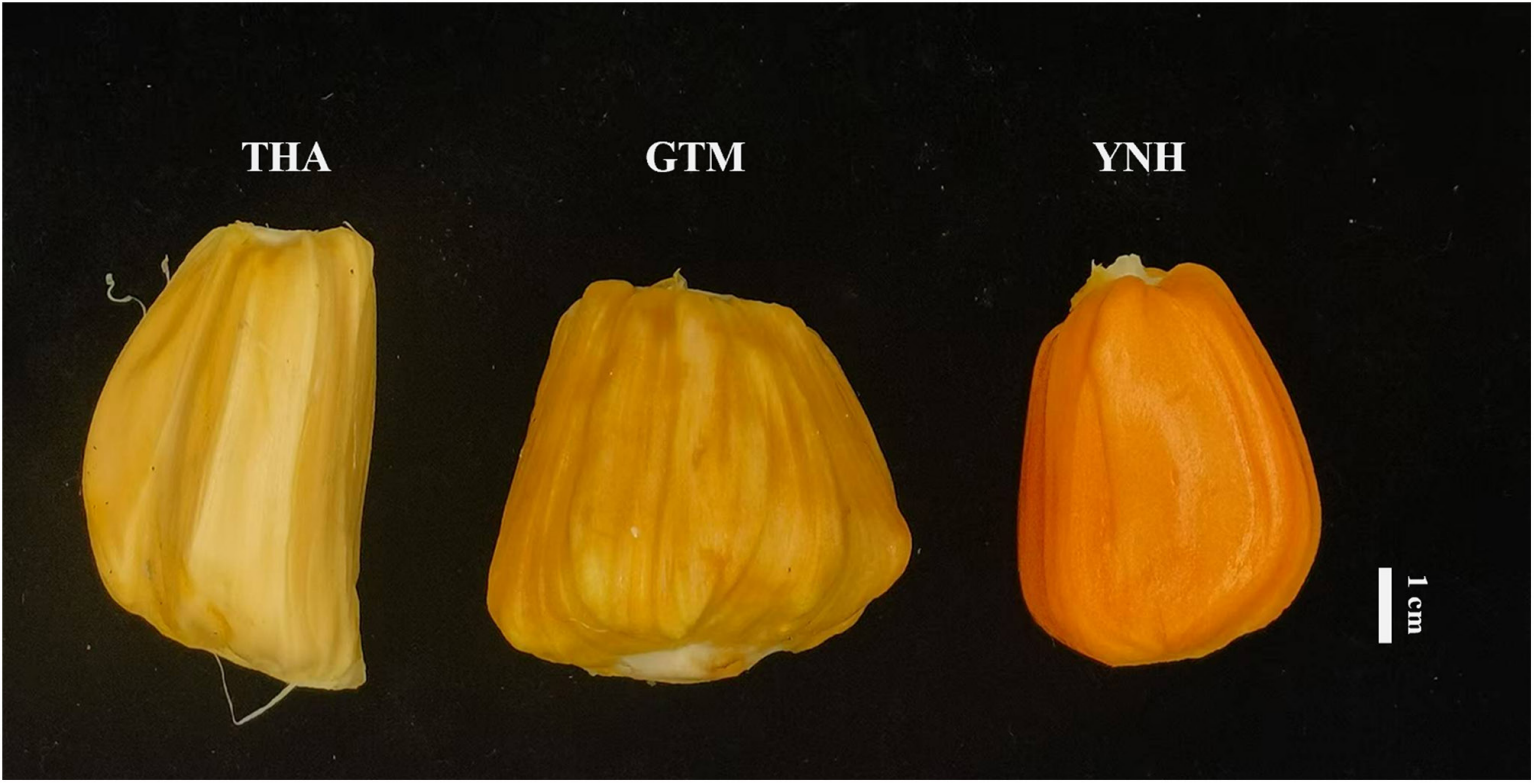
Color phenotype of three jackfruit cultivars. THA (left), GTM (middle), and YNH (right). Scale bar: 1 cm.

Three jackfruit cultivars were harvested from the jackfruit germplasm resource nursery, and the pulps of THA, GTM, and YNH were imaged (Figure 1). THA was light yellow, GTM was yellow, and YNH was orange. As shown in Table 1, the pulp of THA exhibited the highest L* value, indicating the greatest lightness. The pulp of YNH had the maximum a* value, thus showing a stronger red tendency compared to other cultivars. Regarding the b* value, the pulp of GTM had the lowest, consequently, the pulps of THA and YNH showed a more yellowish tendency compared to that of GTM.

**TABLE 1:** Comparison of the pulp color from three cultivars.

Cultivar	L*	a*	b*
THA	77.79 ± 1.13	16.53 ± 0.86	45.04 ± 1.04
GTM	69.40 ± 1.15	18.34 ± 0.48	42.22 ± 0.61
YNH	62.34 ± 0.68	34.16 ± 0.69	45.36 ± 0.79

### 3.2 DAMs Analysis

To explore the potential chemical components contributing to pulp coloration in different jackfruit cultivars, metabolomic analysis was conducted using UPLC‐MS/MS. Pearson's correlation coefficient showed good reproducibility among the three biological replicates (Figure S1a). A total of 1381 metabolites were identified in these pulp samples, including amino acids and derivatives, lipids, flavonoids, phenolic acids, alkaloids, and terpenoids (Table 2).

**TABLE 2:** Types of metabolites identified in the pulps of three jackfruit cultivars.

Class I	Percentage (%)	Class II
Amino acids and derivatives	14.77	—
Lipids	13.98	Free fatty acids, glycerol ester, LPE, LPC, sphingolipids and etc.
Flavonoids	11.01	Flavones, flavonols, flavanonols, flavanones, flavanols, isoflavones, chalcones, anthocyanidins and etc.
Phenolic acids	10.79	—
Alkaloids	10.28	Alkaloids, plumerane, phenolamine, pyridine alkaloids and etc.
Terpenoids	9.12	Sesquiterpenoids, monoterpenoids, ditepenoids and etc.
Organic acids	4.20	—
Lignans and coumarins	4.06	Lignans and coumarins
Nucleotides and derivatives	3.69	—
Quinones	0.72	Anthraquinone, quinones and etc.
Steroids	0.58	Steroidal saponins, steroid and etc.
Tannins	0.51	Proanthocyanidins and tannin
Others	16.29	Saccharides, vitamin, alcohol compounds, aldehyde compounds and etc.

**FIGURE 2:**
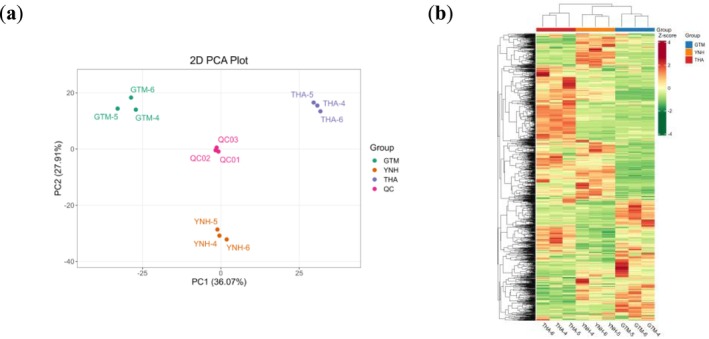
Metabolites analysis of THA, GTM, and YNH. (a) Principal component analysis of the samples. (b) Accumulation pattern of DAMs.

PCA showed that the first principal component explained 36.07% of the total variance, while the second principal component explained 27.91% (Figure 2a). The metabolites were clearly separated among the three jackfruit pulp samples. Hierarchical cluster analysis further classified the accumulation and variation patterns of metabolites across samples (Figure 2b). In the heatmap, metabolites in the pulps of YNH and GTM showed similar accumulation trends.

**FIGURE 3:**
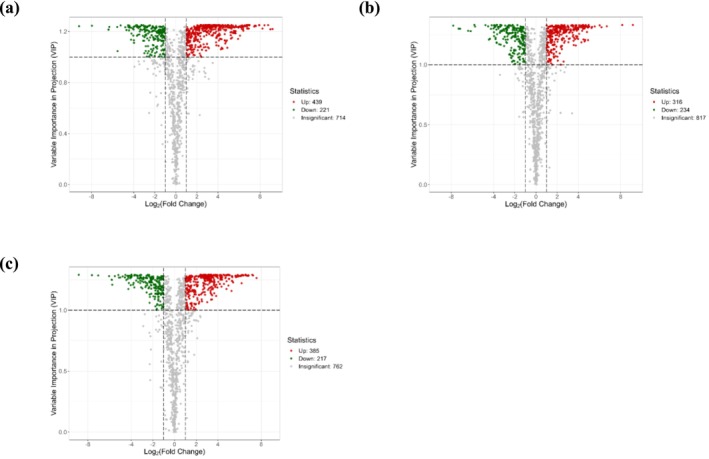
Volcano plots of DAMs in different comparison groups. (a) THA vs. GTM, (b) THA vs. YNH, and (c) YNH vs. GTM.

Comparative analysis identified 660, 550, and 602 DAMs in the groups THA vs. GTM, THA vs. YNH, and YNH vs. GTM, respectively (Figure 3, |log2 FC| ≥ 1, and VIP ≥ 1). Between THA vs. GTM, 439 DAMs were upregulated and 221 were downregulated, including flavonoids, lipids, and amino acids and derivatives (Figure 3a). Between THA vs. YNH, 316 upregulated and 234 downregulated DAMs, including amino acids and derivatives, lipids, flavonoids, and terpenoids (Figure 3b). Between YNH vs. GTM, 385 upregulated and 217 downregulated DAMs, including flavonoids, amino acids and derivatives, and phenolic acids (Figure 3c).

### 3.3 KEGG Enrichment of DAMs

**FIGURE 4:**
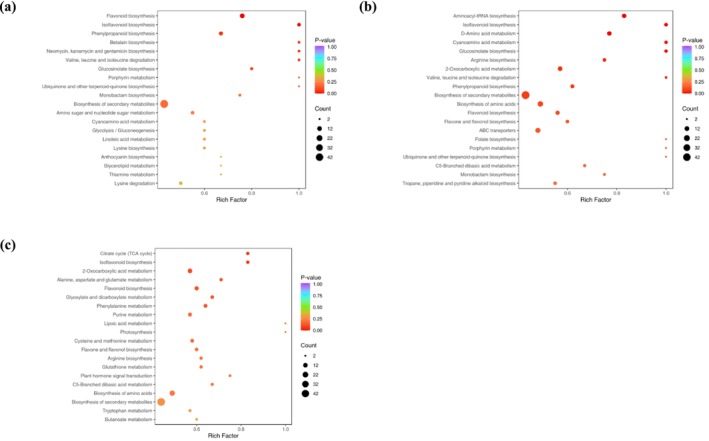
KEGG enrichment analysis of DAMs in different comparison groups. (a) THA vs. GTM, (b) THA vs. YNH, and (c) YNH vs. GTM. The y‐axis represents the enriched pathway, and the x‐axis represents the enrichment factor (number of DAMs in a pathway divided by the total number of DAMs). The size of the dot indicates the number of DAMs, with redder colors corresponding to smaller *p*‐values.

KEGG enrichment analysis revealed that DAMs between THA vs. GTM are enriched in flavonoid biosynthesis (ko00941); isoflavonoid biosynthesis (ko00943); phenylpropanoid biosynthesis (ko00940); valine, leucine, and isoleucine degradation (ko00280); betalain biosynthesis (ko00965); and neomycin, kanamycin, and gentamicin biosynthesis (ko00524) (Figure 4a). In THA vs. YNH, significant enrichment was observed in aminoacyl‐tRNA biosynthesis (ko00970), isoflavonoid biosynthesis (ko00943), D‐amino acid metabolism (ko00470), cyanoamino acid metabolism (ko00460), and glucosinolate biosynthesis (ko00966) (Figure 4b). In YNH vs. GTM, DAMs were enriched in the citrate cycle (ko00020); isoflavonoid biosynthesis (ko00943); 2‐Oxocarboxylic acid metabolism (ko01210); alanine, aspartate, and glutamate metabolism (ko00250); and flavonoid biosynthesis (ko00941) (Figure 4c). These results indicate that some of the flavonoid‐related pathways (ko00943 and ko00941) and their potential metabolites may play key roles in jackfruit pulp coloration.

### 3.4 Differentially Accumulated Flavonoids (DAFs) in Jackfruits

**FIGURE 5:**
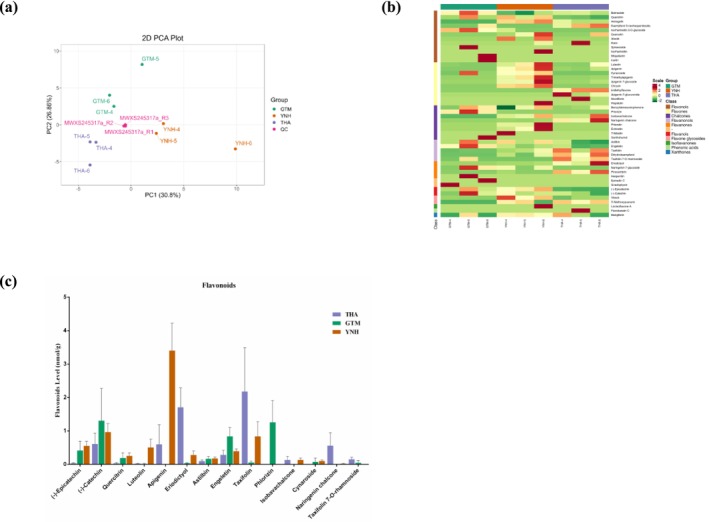
Multivariate analysis and contents of flavonoids in jackfruit pulps. (a) Principal component analysis (PCA) of flavonoids, (b) hierarchical clustering of flavonoid accumulation patterns, and (c) flavonoid levels in THA, GTM, and YNH. The error line represents the standard deviation (*n* = 3).

Forty‐eight flavonoids were detected in the jackfruit pulp samples, including 12 flavonols, 10 flavones, 9 chalcones, 5 flavanonols, 4 flavanones, 2 flavanols, 2 flavone glycosides, 1 xanthone, and 1 isoflavanone (Table 3, Table S1). PCA analysis revealed that the first principal component accounted for 30.8% of the total variance, distinguishing samples based on orange flesh (YNH) and yellow flesh (GTM, THA) (Figure 5a). The second principal component explained 26.86% of the total variance. Hierarchical cluster analysis revealed distinct accumulation trends of flavonoids among the cultivars (Figure 5b). Fourteen flavonoids with relatively higher contents were highlighted in a histogram (Figure 5c). Among them, apigenin (3.40 nmol/g) detected in YNH pulp was the most abundant, followed by taxifolin (2.18 nmol/g) and eriodictyol (1.71 nmol/g) in THA pulp, and (‐)‐catechin (1.31 nmol/g) and phlorizin (1.26 nmol/g) in GTM pulp.

**TABLE 3:** Flavonoids detected in the pulps of the three jackfruit cultivars.

No.	Compounds	Class	Mean (THA) (nmol/g)	Mean (GTM) (nmol/g)	Mean (YNH) (nmol/g)
1	Phlorizin	Chalcones	0.000170788567	1.25777647	0.00324509516
2	Naringenin chalcone	Chalcones	0.561979745	0	0.0176662741
3	Isobavachalcone	Chalcones	0.132942919	0	0.131350438
4	Echinatin	Chalcones	0	0	0.0265768119
5	Xanthohumol	Chalcones	0	0.00592024473	0
6	Benzylideneacetophenone	Chalcones	0.00347540393	0.00498761103	0.00374152266
7	Phloretin	Chalcones	0	0	0.00330437223
8	Trilobatin	Chalcones	0	0	0.000479935837
9	Flavokawain C	Chalcones	0.00017608064	0	0
10	(‐)‐Catechin	Flavanols	0.611104732	1.30656862	0.966517401
11	(‐)‐Epicatechin	Flavanols	0.0338430544	0.41442699	0.553934802
12	Eriodictyol	Flavanones	1.709763945	0.0371273369	0.280053779
13	Pinocembrin	Flavanones	0.0832716693	0	0.00308150465
14	Naringenin‐7‐glucoside	Flavanones	0	0.0508867419	0.021850257
15	Hesperidin	Flavanones	0	0.00663879903	0
16	Taxifolin	Flavanonols	2.17752044	0.0542518938	0.835326787
17	Engeletin	Flavanonols	0.284616011	0.835382203	0.394046816
18	Taxifolin 7‐O‐rhamnoside	Flavanonols	0.1498512	0.0437895423	0
19	Astilbin	Flavanonols	0.09977943	0.167775681	0.177255011
20	Dihydrokaempferol	Flavanonols	0.0351994411	0	0.00959131823
21	3′‐Methoxypuerarin	Flavone glycosides	0.0107739316	0	0.00686881093
22	Vitexin	Flavone glycosides	0.00207300014	0.00206535228	0.0166207354
23	Apigenin	Flavones	0.597555581	0.00683781408	3.402291575
24	Cynaroside	Flavones	0	0.0755198893	0.104103357
25	Luteolin	Flavones	0.021028076	0.0136597053	0.5066963645
26	Chrysin	Flavones	0	0	0.0376574029
27	6‐Methylflavone	Flavones	0.0164837193	0	0
28	Apigenin‐7‐glucuronide	Flavones	0.00206461133	0	0
29	Nicotiflorin	Flavones	0.00224048319	0	0
30	Hispidulin	Flavones	0	0	0.00888504357
31	Apigenin 7‐glucoside	Flavones	0.000750598313	0	0.0172158722
32	Trimethylapigenin	Flavones	0.00026183626	0	0.0190452468
33	Quercitrin	Flavonols	0.034945625	0.188351171	0.255167046
34	Baimaside	Flavonols	0.0333940366	0.0553005291	0.0184880208
35	Astragalin	Flavonols	0.0169248755	0	0.0445409721
36	Quercetin	Flavonols	0.0089542872	0	0.0197735372
37	Rutin	Flavonols	0.006603175	0	0.00129449016
38	Isorhamnetin 3‐O‐glucoside	Flavonols	0	0.00608819254	0.000700744307
39	Kaempferol 3‐neohesperidoside	Flavonols	0.0075388824	0.00202239662	0
40	Spiraeoside	Flavonols	0	0.00419022877	0
41	Icariin	Flavonols	0	0.00333364557	0
42	Miquelianin	Flavonols	0	0.00260764557	0
43	Isorhamnetin	Flavonols	0	0	0.00066452888
44	Afzelin	Flavonols	0	0	0.000398565661
45	Licoisoflavone A	Isoflavanones	0	0	0.000260333979
46	Mangiferin	Xanthones	0.00336509721	0.00203792484	0.00523099578
47	Epmedin C	—	0	0.124053165	0
48	Sciadopitysin	—	0	0.00252066993	0

**FIGURE 6:**
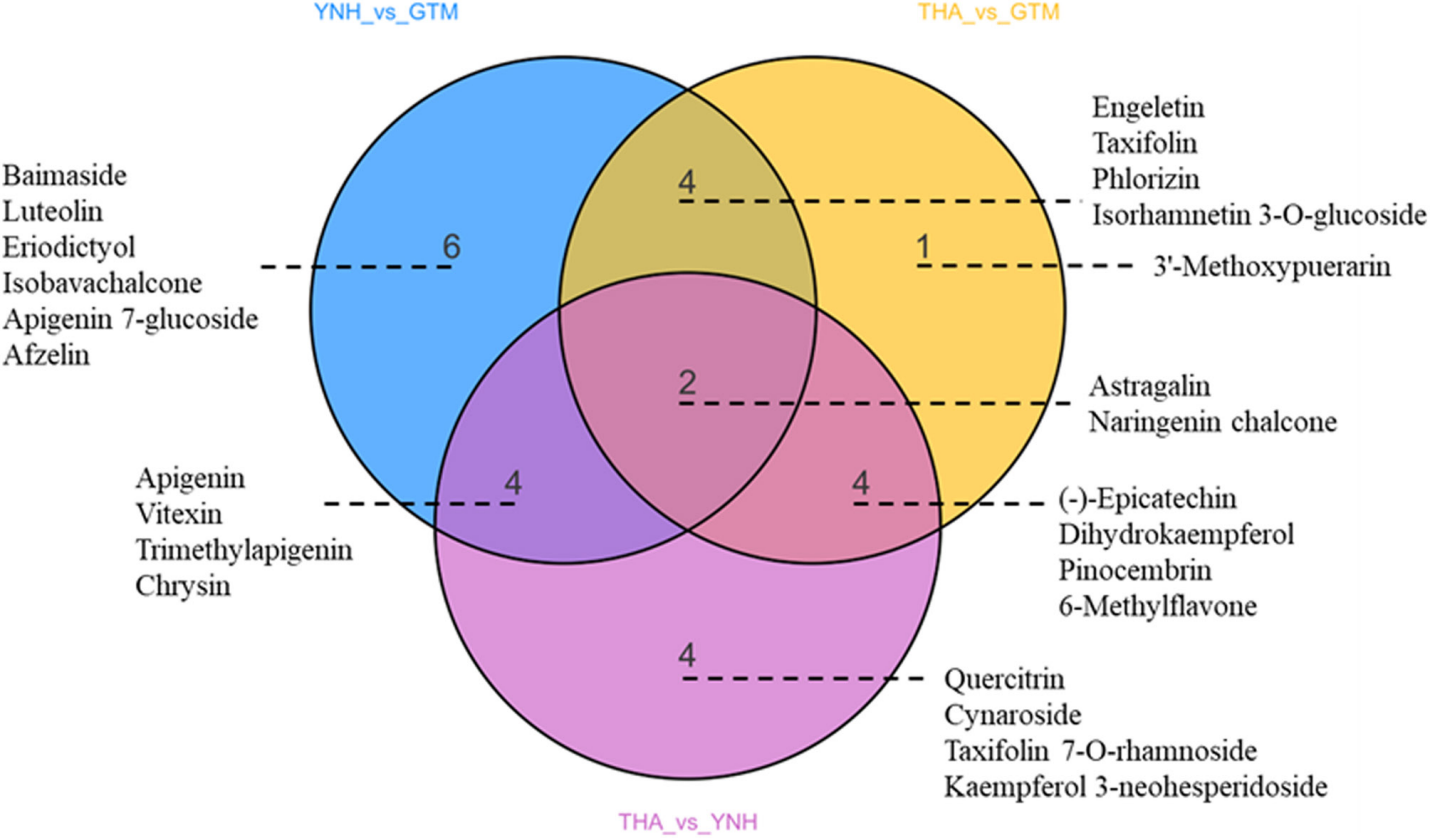
Venn diagram analysis of DAFs in THA vs. GTM, THA vs. YNH, and YNH vs. GTM.

Comparative analysis identified 25 DAFs (FC ≥ 2 or ≤ 0.5, and VIP ≥ 1). Among these, 10 flavonoids predominantly accumulated in THA pulp, 4 in GTM pulp, and 11 in YNH pulp (Table S1). Specifically, 11 DAFs were identified in THA vs. GTM, 14 in THA vs. YNH, and 16 in YNH vs. GTM, with astragalin and naringenin chalcones being common DAFs among all comparison groups (Figure 6). Phlorizin accumulated significantly in GTM, with log2FC values of −12.85 and −8.60 in the THA vs. GTM and YNH vs. GTM groups, respectively, suggesting that it is a major contributor to the yellow tone of GTM pulp. Apigenin significantly accumulated in YNH with a log2FC value of 9.48 and −3.03 between YNH vs. GTM and THA vs. YNH groups, respectively, indicating its potential role in distinguishing orange from yellow flesh. Naringenin chalcone significantly accumulated in THA with a mean content of 0.56 nmol/g, suggesting that naringenin chalcone was a major contributor to the light yellow tone of THA pulp (Table S1). Besides, (‐)‐epicatechin accumulated in the pulps of GTM and YNH, taxifolin accumulated in the pulps of THA and YNH, and luteolin accumulated in the pulp of YNH (mean content of 0.51 nmol/g).

### 3.5 Differentially Accumulated Carotenoids (DACs) in Jackfruits

**FIGURE 7:**
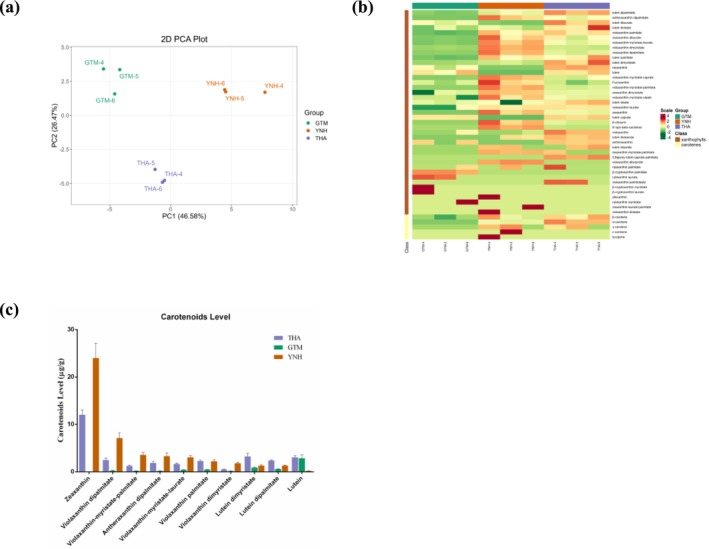
Multivariate analysis and contents of carotenoids in jackfruit pulps. (a) PCA of carotenoids, (b) hierarchical clustering of carotenoid accumulation patterns, and (c) carotenoid levels in THA, GTM, and YNH. The error line represents the standard deviation (*n* = 3).

In this study, 46 carotenoids were detected in the samples from the three cultivars, comprising 5 carotenes and 41 xanthophylls, including 1 lutein and 9 derivatives, 1 violaxanthin and 12 derivatives, 1 zeaxanthin and 3 derivatives, and 3 *β*‐cryptoxanthin derivatives (Table 4, Table S2). PCA revealed that the first and second principal components accounted for 46.58% and 26.47% of the total variance, respectively (Figure 7a). Hierarchical cluster analysis showed that GTM had a relatively low carotenoid content (Figure 7b). Ten carotenoids with relatively high contents in the histogram were selected (Figure 7c). Zeaxanthin (24.00 μg/g) detected in the YNH pulp was the highest, followed by zeaxanthin (12.04 μg/g) detected in the THA pulp and violaxanthin dipalmitate (7.13 μg/g) detected in the YNH pulp. In contrast, only lutein (2.88 μg/g) showed a relatively high accumulation in the pulp of GTM. The esterified forms of violaxanthin accumulated abundantly in the pulp of both THA and YNH.

**TABLE 4:** Carotenoids detected in the pulps of the three jackfruit cultivars.

No.	Compounds	Class	Mean (THA) (μg/g)	Mean (GTM) (μg/g)	Mean (YNH) (μg/g)
1	*β*‐carotene	Carotenes	0.913313252	0.624712206	0.838434664
2	*α*‐carotene	Carotenes	0.855545831	0	0.362564374
3	*γ*‐carotene	Carotenes	0.149877373	0	0.18195553
4	*ε*‐carotene	Carotenes	0	0	0.0096414106
5	Lycopene	Carotenes	0	0	0.23844309
6	Lutein	Xanthophylls	3.04749412	2.87983338	0.214578795
7	Lutein dilaurate	Xanthophylls	0.599265677	0.374274026	0.144188586
8	Lutein dioleate	Xanthophylls	0.14916742	0.115242105	0.122321318
9	Lutein dipalmitate	Xanthophylls	2.36488051	0.574486186	1.30640446
10	Lutein palmitate	Xanthophylls	0.661078799	0.235002174	0.643163945
11	Lutein dimyristate	Xanthophylls	3.24208491	0.871146015	1.31539872
12	Lutein oleate	Xanthophylls	0.131253346	0.0879684023	0.0672199178
13	Lutein caprate	Xanthophylls	0.113350205	0.10026361	0
14	Lutein distearate	Xanthophylls	0.0941233691	0	0.0521462409
15	Lutein stearate	Xanthophylls	0.0600981256	0	0.0560128534
16	Antheraxanthin	Xanthophylls	0.358371178	0.227388641	0
17	Antheraxanthin dipalmitate	Xanthophylls	1.85800962	0.216578987	3.29958206
18	Violaxanthin	Xanthophylls	0.678395594	0.134873545	0.0500955557
19	Violaxanthin‐myristate‐palmitate	Xanthophylls	1.25372973	0.231133726	3.57896538
20	Violaxanthin palmitate	Xanthophylls	2.25088105	0.458696344	2.19681407
21	Violaxanthin dilaurate	Xanthophylls	0.304230816	0.118456713	0.452666444
22	Violaxanthin‐myristate‐laurate	Xanthophylls	1.64108417	0.402613129	3.05690693
23	Violaxanthin dimyristate	Xanthophylls	0.505543186	0.184138887	1.80040749
24	Violaxanthin dipalmitate	Xanthophylls	2.49448471	0.256115776	7.13282596
25	Violaxanthin‐myristate‐caprate	Xanthophylls	0.311824365	0.139903623	0.603393298
26	Violaxanthin‐myristate‐oleate	Xanthophylls	0.0961440211	0.0524080787	0.155849044
27	Violaxanthin laurate	Xanthophylls	0.0879180691	0.0256067844	0.0862634338
28	Violaxanthin dibutyrate	Xanthophylls	0	0	0.0807190713
29	Violaxanthin palmitoleate	Xanthophylls	0.0508836497	0	0
30	Violaxanthin dioleate	Xanthophylls	0	0	0.0256831138
31	Neoxanthin	Xanthophylls	0.185786784	0.135712326	0.0962217525
32	Fucoxanthin	Xanthophylls	0.233124628	0.238849121	0.274623332
33	Zeaxanthin	Xanthophylls	12.0371065	0	23.99531405
34	Zeaxanthin dimyristate	Xanthophylls	0.0830590997	0.0542147492	0.127888112
35	Zeaxanthin‐myristate‐palmitate	Xanthophylls	0.0241902259	0	0.0822821773
36	Zeaxanthin‐laurate‐palmitate	Xanthophylls	0	0	0.0292092929
37	*β*‐citraurin	Xanthophylls	0.0265425885	0	0.0925662336
38	8′‐apo‐beta‐carotenal	Xanthophylls	0.0445557963	0	0.439004518
39	*β*‐cryptoxanthin palmitate	Xanthophylls	0	0.104042115	0
40	*β*‐cryptoxanthin myristate	Xanthophylls	0	0.0273529057	0
41	*β*‐cryptoxanthin laurate	Xanthophylls	0	0.0309981902	0
42	Alloxanthin	Xanthophylls	0	0	0.0000262584038
43	5,6epoxy‐lutein‐caprate‐palmitate	Xanthophylls	0.5094605	0	0
44	Rubixanthin palmitate	Xanthophylls	0.069372469	0.035218587	0.0471832603
45	Rubixanthin palmitate	Xanthophylls	0	0.0533207331	0
46	Rubixanthin palmitate	Xanthophylls	0	0.0267331393	0

**FIGURE 8:**
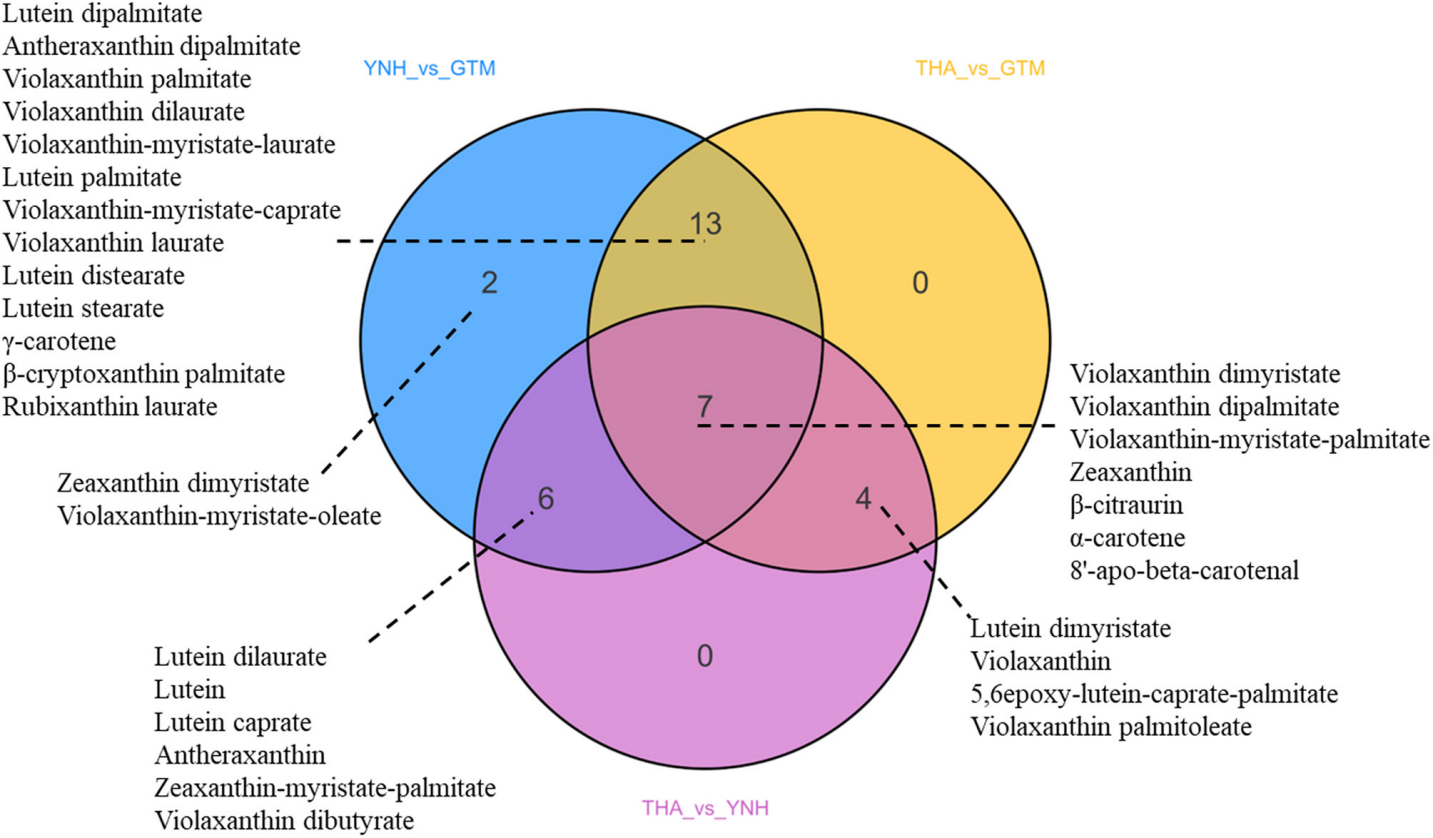
Venn diagram of DACs in THA vs. GTM, THA vs. YNH, and YNH vs. GTM.

Comparative analysis identified 32 DACs (FC ≥ 2 or ≤ 0.5 and VIP ≥ 1). Of these, 15 carotenoids predominantly accumulated in THA, 2 in GTM, and 15 in YNH (Table S2). Specifically, 24 DACs were observed in THA vs. GTM, 17 in THA vs. YNH, and 28 in YNH vs. GTM (Figure 8), with 7 carotenoids—violaxanthin dimyristate, violaxanthin dipalmitate, violaxanthin‐myristate‐palmitate, zeaxanthin, *β*‐citraurin, *α*‐carotene, and 8′‐apo‐beta‐carotenal—common to all comparisons. Zeaxanthin, violaxanthin palmitate, violaxanthin‐myristate‐laurate, antheraxanthin dipalmitate, and lutein dipalmitate were significantly accumulated in THA and YNH. Lutein significantly accumulated in THA and GTM, indicating its potential role in the yellow tone. Other carotenoids, including lutein dimyristate, violaxanthin, and 5,6 epoxy‐lutein‐caprate‐palmitate significantly accumulated in THA. Violaxanthin dipalmitate, violaxanthin‐myristate‐palmitate, and 8′‐apo‐beta‐carotenal significantly accumulated in YNH.

### 3.6 Transcriptome Sequencing and Annotation

**FIGURE 9:**
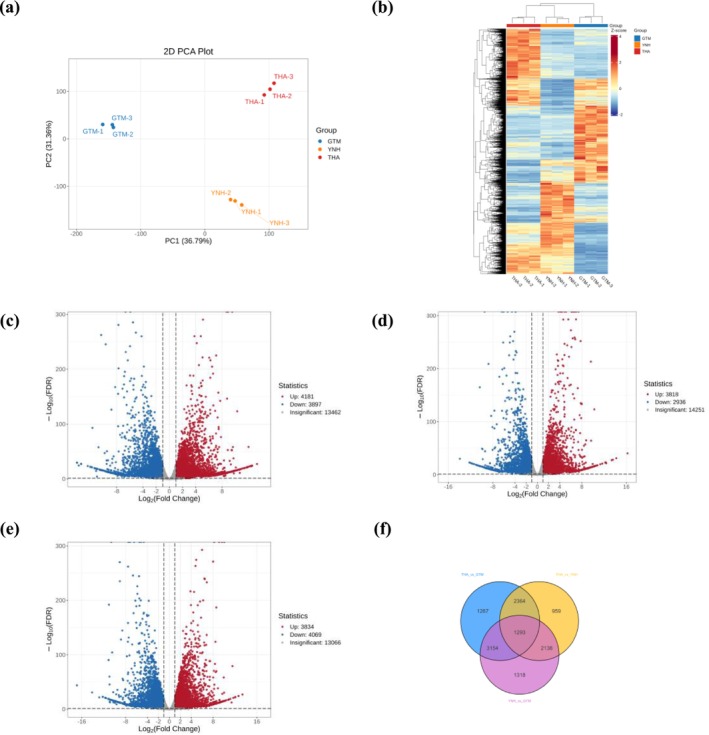
Transcriptome analysis of jackfruit pulps. PCA of samples (a), and hierarchical clustering of DEGs expression patterns (b) of THA, GTM, and YNH. Number of up‐ and downregulated DEGs in the comparison groups: THA vs. GTM (c), THA vs. YNH (d), and YNH vs. GTM (e). Venn diagram showing DEGs common and unique to the three comparison groups (f).

Analysis of the base composition indicated that the guanine−cytosine (GC) content of each sample exceeded 45% and the number of clean reads per sample ranged from 4.95 to 6.27 Gb after filtration (Table S3). Pearson's correlation coefficient showed good reproducibility among the three biological replicates (Figure S1b). Reconstruction of the transcripts using the StringTie software identified 4818 novel genes, which are unigenes not included in the reference genome (Lin et al. 2022). PCA results showed that the first and second principal components explained 36.79% and 31.36% of the total variance, respectively (Figure 9a). Hierarchical cluster analysis showed that genes from THA and YNH pulps shared similar expression patterns (Figure 9b). Several key genes, including five salicylic acid 3‐hydroxylase (*S3H*: *AHE.Chr09.1460*, *AHE.Chr09.1461*, *AHE.Chr10.1297*, *AHE.Chr18.739*, *AHE.Chr10.1306*), one 4‐coumarate‐CoA ligase (*4CL*: *AHE.Chr12.601*), one phenylalanine ammonia‐lyase (*PAL*: *AHE.Chr26.1172*), one zeaxanthin epoxidase (*ZEP*: *AHE.Chr16.1073*), one cinnamoyl‐CoA reductase (*CCR*: *AHE.Chr24.1039*) and one flavonol synthase (*FLS*: *AHE.Chr05.909*) were highly expressed in all three jackfruit pulps. Using thresholds of FDR < 0.05 and ¦log2 FC¦ ≥ 1, 8078 DEGs were identified between THA and GTM, 6754 between THA and YNH, and 7903 between YNH and GTM (Figure 9c–e) (Table S4). Among these, 4181, 3818, and 3834 DEGs were upregulated, and 3897, 2936, and 4069 were downregulated in the THA vs. GTM, THA vs. YNH, and YNH vs. GTM comparison groups, respectively. As shown in Figure 9f, 1293 DEGs were common to all three comparisons. Of which, 9 DEGs (*AHE.Chr04.975*, *AHE.Chr10.1299*, *AHE.Chr13.1292*, *AHE.Chr15.1170*, *AHE.Chr16.1036*, *AHE.Chr22.13*, *AHE.Chr23.383*, *AHE.Chr27.485*, and *novel.3787*) were associated with flavonoid biosynthesis, and 12 DEGs (*AHE.Chr01.273*, *AHE.Chr02.742*, *AHE.Chr04.1544*, *AHE.Chr07.1435*, *AHE.Chr07.1525*, *AHE.Chr11.451*, *AHE.Chr16.1073*, *AHE.Chr18.949*, *AHE.Chr21.1556*, *AHE.Chr22.1872*, *novel.4570*, and *novel.2938*) were associated with carotenoid biosynthesis.

KEGG and GO enrichment analyses were performed to assess the biological functions of the DEGs. In THA vs. GTM, 3052 DEGs were annotated in 148 KEGG pathways (Table S4), with the top five enriched KEGG pathways being carbon metabolism, steroid biosynthesis, amino acid biosynthesis, fatty acid biosynthesis, and glycolysis/gluconeogenesis (Figure S2). Moreover, carotenoid biosynthesis (ko00906, 30 DEGs [15 upregulated, 15 downregulated]), flavonoid biosynthesis (ko00941, 20 DEGs [8 upregulated, 12 downregulated]), isoflavonoid biosynthesis (ko00943, 6 DEGs [2 upregulated, 4 downregulated]) and flavone and flavonol biosynthesis (ko00944, 5 DEGs [3 upregulated, 2 downregulated]) were also significantly enhanced. GO clustering analysis classified DEGs into cellular component (2 terms), biological process (22 terms), and molecular function (19 terms) (Figure S3).

In THA vs. YNH, 2489 DEGs were annotated in 145 KEGG pathways (Table S4). The top five enriched KEGG pathways included ether lipid metabolism; steroid biosynthesis; protein processing in the endoplasmic reticulum; glycerophospholipid metabolism; and alanine, aspartate, and glutamate metabolism (Figure S4). Moreover, Carotenoid biosynthesis (ko00906, 23 DEGs [19 upregulated, 4 downregulated]), flavonoid biosynthesis (ko00941, 21 DEGs [4 upregulated, 17 downregulated]), isoflavonoid biosynthesis (ko00943, 8 DEGs [3 upregulated, 5 downregulated]), and flavone and flavonol biosynthesis (ko00944, 6 DEGs [4 upregulated, 2 downregulated]) were also significantly enriched. GO enrichment analysis classified the DEGs into cellular component (2 terms), biological process (22 terms), and molecular function (20 terms) (Figure S5).

In YNH vs. GTM, 3031 DEGs were annotated in 148 KEGG pathways (Table S4). The top five enriched KEGG pathways included carbon metabolism, amino acid biosynthesis, citrate cycle (TCA cycle), lipoic acid metabolism, and protein processing in the endoplasmic reticulum (Figure S6). Moreover, carotenoid biosynthesis (ko00906, 23 DEGs [5 upregulated, 18 downregulated]), flavonoid biosynthesis (ko00941, 23 DEGs [15 upregulated, 8 downregulated]), isoflavonoid biosynthesis (ko00943, 9 DEGs [5 upregulated, 4 downregulated]) and flavone and flavonol biosynthesis (ko00944, 6 DEGs [3 upregulated, 3 downregulated]) were also significantly enhanced. GO enrichment analysis classified the DEGs into cellular component (2 terms), biological process (22 terms), and molecular function (20 terms) (Figure S7).

### 3.7 Analysis of Structural Genes Involved in Flavonoid Biosynthesis

The transcript levels of 42 candidate structural genes related to flavonoid biosynthesis were analyzed. Among them, *FLS* (*AHE.Chr05.909*) and *PAL* (*AHE.Chr26.1172*) showed the highest expression in pulp samples, followed by *4CL* (*AHE.Chr28.135*) in THA, *ANS* (*AHE.Chr06.597*) in GTM, *4CL* (*AHE.Chr28.135*) and *HCT* (*AHE.Chr23.12*) in YNH, and *HCT* (*AHE.Chr13.1292*) in GTM. Compared with THA, two *4CL* (*AHE.Chr04.977*, *AHE.Chr20.345*), one *ANS* (*AHE.Chr06.597*), one *F3H* (*AHE.Chr10.1299*), two *C4H* (*AHE.Chr19.24*, *AHE.Chr20.25*), and one *HCT* (*AHE.Chr15.1170*, *AHE.Chr04.1699*) were highly expressed in GTM. Compared with YNH, the expression of one *HCT* (*AHE.Chr13.1292*) and one *DFR* (*AHE.Chr05.1351*) was stronger in THA, whereas the opposite was observed for three *HCTs* (*AHE.Chr23.11*, *AHE.Chr23.12*, *AHE.Chr15.1170*), one *CHI* (*AHE.Chr27.485*), one *F3H* (*AHE.Chr10.1299*), one *ANR* (*novel.3787*), and one *C4H* (*AHE.Chr19.24*, *AHE.Chr20.25*). Compared with GTM, the expression of three *HCTs* (*AHE.Chr15.1170*, *AHE.Chr23.11*, *AHE.Chr23.12*, *AHE.Chr04.1699*), one *ANR* (*novel.3787*), and one *CHI* (*AHE.Chr27.485*) were stronger in YNH, while the opposite was observed for one *ANS* (*AHE.Chr06.597*), two *4CL* (*AHE.Chr04.977*, *AHE.Chr20.345*), and one *HCT* (*AHE.Chr13.1292*). The expression of *ANS* (*AHE.Chr06.597*) was extremely low in THA and YNH, but high in GTM, indicating its key role in GTM pulp coloration.

**FIGURE 10:**
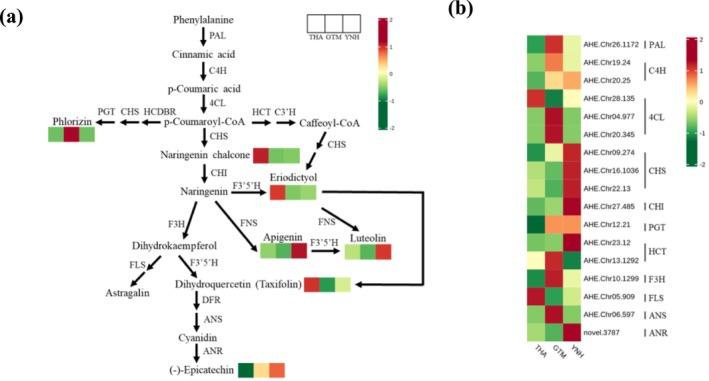
Heatmaps and regulatory network of flavonoid biosynthesis in three jackfruit cultivars. (a) Regulatory network (Shen et al. 2022) of predicted flavonoid biosynthesis in three jackfruit cultivars with different pulp colors. The color scale, ranging from green to red for the heatmap, represents the z‐score value of the flavonoids content in THA, YNH, and GTM. (b) Heatmap showing the z‐score value of the expression levels (FPKM) of the DEGs. Red indicates high expression, and green indicates low expression. *PAL*, phenylalanine ammonia lyase; *C4H*, cinnamic acid 4‐hydroxylase; *4CL*, 4‐coumarate CoA ligase; *CHS*, chalcone synthase; *CHI*, chalcone isomerase; *FNS*, flavone synthase; *HCDBR*, hydroxycinnamoyl‐CoA double bond reductase; *PGT*, phlorizin synthase; *HCT*, shikimate O‐hydroxycinnamoyl transferase; *C3*′*H*, 5‐O‐(4‐coumaroyl)‐D‐quinate 3′‐monooxygenase; *F3*′*5*′*H*, flavonoid 3′,5′‐hydroxylase; *F3H*, flavanone 3‐hydroxylase; *FLS*, flavonol synthesis; *DFR*, dihydroflavonol 4‐reductase; *ANS*, anthocyanidin synthase; *ANR*, anthocyanin reductase.

To further investigate the relationship between the flavonoid‐related genes and metabolites, a pathway integrating structural gene expression and DAFs was constructed (Figure 10a,b). Seven DAFs and 17 DEGs were selected to demonstrate significant differences in flavonoid biosynthesis among THA, GTM, and YNH pulps. Upstream precursors in the flavonoid biosynthesis pathway, including naringenin chalcone, eriodictyol, and taxifolin, accumulated significantly in THA. Phlorizin was the only flavonoid enriched in GTM pulp, while apigenin and its downstream product luteolin were most abundant in YNH. The significant difference in apigenin content among the samples suggests its role in deepening flesh color from yellow to orange. Gene expression analysis revealed that two DEGs (one *4CL* and one *FLS*) were upregulated in THA, eight DEGs (one *PAL*, one *C4H*, two *4CL*, one *PGT*, one *HCT*, one *F3H*, and one *ANS*) in GTM, and eight DEGs (one *C4H*, three *CHS*, one *CHI*, one *PGT*, one *HCT*, and one *ANR*) in YNH (Figure 10b). Two highly expressed genes (*AHE.Chr26.1172* and *AHE.Chr28.135*) upstream of the flavonoid biosynthesis pathway were enriched in GTM and THA, respectively. The gene encoding synthases for the key precursor, *CHI* (*AHE.Chr27.485*), was highly expressed in YNH, while downstream genes *F3H* (*AHE.Chr10.1299*) and *ANS* (*AHE*.*Chr06.597*) were highly expressed in GTM, and one *ANR* (*novel.3787*) was highly expressed in YNH. These findings indicated that flavonoid biosynthesis in THA pulp mainly occurs upstream of the pathway, whereas GTM exhibits flavonoid biosynthesis throughout the pathway.

Co‐expression network analysis of DEGs and DAFs confirmed the role of specific flavonoids in the coloration of jackfruit pulps (Pearson correlation coefficient > 0.8 or < − 0.8, *p*‐value < 0.05; Table S5). In THA vs. GTM, DAFs such as (‐)‐epicatechin, phlorizin, naringenin chalcone, dihydrokaempferol, and astragalin correlated with different pulp colors, with associated DEGs including one *HCT* (*AHE.Chr.04.1699*), one *ANS* (*AHE.Chr.06.597*), one *DFR* (*AHE.Chr.05.1351*), one *FLS* (*AHE.Chr.05.909*), one *FG3* (*AHE.Chr.23.1318*), and two *C4H* (*AHE.Chr.20.25*, *AHE.Chr.19.24*). In THA vs. YNH, DAFs such as (‐)‐epicatechin, apigenin, vitexin, naringenin chalcone, dihydrokaempferol, and astragalin were related to their different pulp colors. The DEGs included one *C4H* (*AHE.Chr.20.25*), one *CHI* (*AHE.Chr.27.485*), one *ANR* (*novel.3787*), one *DFR* (*AHE.Chr.05.1351*), and three *HCT* (*AHE.Chr.23.11*, *AHE.Chr.23.12*, *AHE.Chr.15.1170*). In YNH vs. GTM, DAFs related to different pulp colors included luteolin, apigenin, vitexin, phlorizin, baimaside, and astragalin, and the genes associated with flavonoid biosynthesis included one *ANR* (*novel.3787*), one *CHI* (*AHE.Chr.27.485*), one *ANS* (*AHE.Chr.06.597*), one *FG3* (*AHE.Chr.23.1318*), and four *HCT* (*AHE.Chr.23.11*, *AHE.Chr.23.12*, *AHE.Chr.15.1170*, *AHE.Chr.04.1699)*. These findings indicated that the changes in (‐)‐epicatechin and naringenin chalcones content distinguish the color of THA pulp from that of GTM and YNH pulps. Phlorizin determines the yellow tone of the GTM pulp, and apigenin content distinguishes YNH from THA and GTM. The key DEGs driving these differences in pulp coloration included one *ANR* (*novel.3787*), one *ANS* (*AHE.Chr.06.597*), one *DFR* (*AHE.Chr.05.1351*), one *FLS* (*AHE.Chr.05.909*), one *FG3* (*AHE.Chr.23.1318*), one *CHI* (*AHE.Chr.27.485*), one *C4H* (*AHE.Chr.19.24*), and three *HCTs* (*AHE.Chr.23.11*, *AHE.Chr.23.12*, *AHE.Chr.04.1699*).

### 3.8 Analysis of Structural Genes Involved in Carotenoid Biosynthesis

The transcript levels of 45 candidate structural genes related to carotenoid biosynthesis were analyzed. Among them, *ZEP* (*AHE.Chr16.1073*) showed the highest expression in GTM pulp, followed by *VDE* (*AHE.Chr01.273*) and *PSY* (*AHE.Chr22.1872*) in THA pulp. Compared with GTM, one *PSY* (*AHE.Chr22.1872*), one *VDE* (*AHE.Chr01.273*), one *ZDS* (*AHE.Chr12.485*), one *PDS* (*AHE.Chr15.1439*), one *LCYE* (*AHE.Chr03.431*), one *ZEP* (*AHE.Chr02.742*), and three *CCD1* (*AHE.Chr08.2108*, *AHE.Chr22.1621*, *novel 2938*) were highly expressed in THA, while the opposite was observed for one *ZEP* (*AHE.Chr16.1073*) and one *CCD1* (*novel.2933*). Compared with YNH, one *PSY* (*AHE.Chr22.1872*), one *VDE* (*AHE.Chr01.273*), one *PDS* (*AHE.Chr15.1439*), one *LUT5* (*AHE.Chr11.451*), and two *CCD1* (*AHE.Chr08.2108*, *novel 2938*) were highly expressed in THA. Compared with YNH, the expression of one *VDE* (*AHE.Chr01.273*), one *ZEP* (*AHE.Chr16.1073*), one *AOG* (*AHE.Chr18.949*), and one *PDS* (*AHE.Chr07.1435*) was higher in the GTM, whereas the opposite was observed for one *LCYE* (*AHE.Chr03.431*).

**FIGURE 11:**
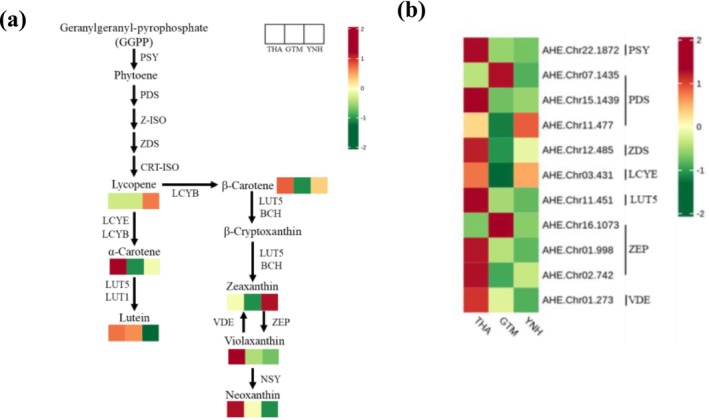
Heatmaps and regulatory network of carotenoid biosynthesis in three jackfruit cultivars. (a) Regulatory network (Walter and Strack 2011) of predicted carotenoid biosynthesis in three jackfruit cultivars with varying pulp colors. The color scale, ranging from green to red for the heatmap, represents the z‐score value of the carotenoid content in THA, YNH, and GTM. (b) Heatmap showing the z‐score value of the expression levels (FPKM) of the DEGs. Red indicates high expression, and green indicates low expression. *PSY*, phytoene synthase; *PDS*, phytoene desaturase; *Z‐ISO*, 15‐cis‐zeta‐carotene isomerase; *ZDS*, zeta‐carotene desaturase; *CRT‐ISO*, carotenoid isomerase; *LCYE*, lycopene *ε*‐cyclase; *LCYB*, lycopene *β*‐cyclase; *LUT*, beta‐ring hydroxylase; *BCH*, *β*‐carotene hydroxylase; *ZEP*, zeaxanthin epoxidase; *VDE*, violaxanthin de‐epoxidase; *NSY*, neoxanthin synthetase.

Based on a previously reported carotenoid biosynthesis pathway (Walter and Strack 2011), we constructed a pathway diagram that included the expression pattern of each structural gene and the DACs (Figure 11a,b). Seven DACs and 11 DEGs were selected to demonstrate significant differences in carotenoid biosynthesis in the THA, GTM, and YNH pulps. THA pulp was enriched in *α*‐carotene, which was associated with yellow‐orange pigments (Khoo et al. 2011), violaxanthin, and neoxanthin (both associated with the yellow tone (Hu et al. 2017)). GTM pulp showed accumulation of only lutein, at levels higher than YNH but lower than THA, while YNH pulp was dominated by zeaxanthin, an orange pigment (Khoo et al. 2011). These findings suggest that lutein contributes to the yellow tone of THA and GTM pulps, whereas zeaxanthin is the main determinant of the orange tone of YNH pulp.

Gene expression analysis showed that eight DEGs (one *PSY*, one *PDS*, one *ZDS*, one *LCYE*, one *LUT5*, two *ZEP*, and one *VDE*) were upregulated in THA pulp, two DEGs (one *PDS*, one *ZEP*) in GTM pulp, and two DEGs (one *PDS* and one *LCYE*) in YNH pulp. *ZEP* (*AHE.Chr.16.1073*) exhibited the highest expression in GTM pulp, whereas *PSY* (*AHE.Chr.22.1872*) and *VDE* (*AHE.Chr.01.273*) were highly expressed in THA pulp. These findings indicate that carotenoid biosynthesis is highly active in THA pulp, whereas YNH showed comparatively lower activity.

Co‐expression network analysis of DEGs and DACs in the three comparison groups confirmed their roles in pulp coloration (Pearson correlation coefficient > 0.8 or < − 0.8, *p*‐value < 0.05; Table S5). In THA vs. GTM, DACs, such as zeaxanthin, *α*‐carotene, and violaxanthin, showed an association with their pulp colors. The DEGs associated with carotenoid biosynthesis in these cultivars mainly included one *PSY* (*AHE.Chr.22.1872*), one *ZDS* (*AHE.Chr.12.485*), one *LCYE* (*AHE.Chr.03.431*), one *LUT5* (*AHE.Chr.11.451*), one *PDS* (*AHE.Chr.15.1439*), and two *ZEPs* (*AHE.Chr.16.1073*, *AHE.Chr.02.742*). In THA vs. YNH, DACs associated with the pulp colors included lutein, zeaxanthin, *α*‐carotene, violaxanthin and antheraxanthin, while DEGs, including one *PSY* (*AHE.Chr.22.1872*), one *LUT5* (*AHE.Chr.11.451*), one *PDS* (*AHE.Chr.15.1439*), and three *ZEPs* (*AHE.Chr.16.1073*, *AHE.Chr.02.742*, *AHE.Chr.01.998*) were identified to be associated with carotenoid biosynthesis. The DACs related to their pulp colors in YNH vs. GTM included lutein, zeaxanthin, *α*‐carotene, and antheraxanthin. The associated genes included one *PSY* (*AHE.Chr.22.1872*), one *ZDS* (*AHE.Chr.12.485*), one *LCYE* (*AHE.Chr.03.431*), one *LUT5* (*AHE.Chr.11.451*), and two *ZEPs* (*AHE.Chr.16.1073*, *AHE.Chr.02.742*). These results indicate that the levels of zeaxanthin and *α*‐carotene are key determinants of pulp color, with one *PSY* (*AHE.Chr.22.1872*), one *ZDS* (*AHE.Chr.12.485*), one *LCYE* (*AHE.Chr.03.431*), one *LUT5* (*AHE.Chr.11.451*), one *PDS* (*AHE.Chr.15.1439*), and two *ZEPs* (*AHE.Chr.16.1073*, *AHE.Chr.02.742*) as the crucial DEGs controlling carotenoid biosynthesis in the three cultivars.

## 4 Discussion

Numerous varieties of jackfruit are found worldwide. They exhibit substantial diversity in pulp color, ranging from off‐white to yellow, dark orange and even red, which correlates with differences in fruit morphology, physiology, and physicochemical properties (Ranasinghe et al. 2019; Morelos‐Flores, Chacón‐López et al. 2024; Morelos‐Flores, Montalvo‐González et al. 2024). In this study, we investigated three cultivars—THA (light yellow), GTM (yellow), and YNH (orange)—to elucidate the metabolic and genetic bases of pulp coloration. Using combined metabolomic and transcriptomic analyses, we identified key flavonoids, carotenoids, and DEGs contributing to color differences.

Metabolomic profiling revealed that amino acids and derivatives, lipids, flavonoids, phenolic acids, alkaloids, and terpenoids are abundant in jackfruit pulp, with significant differences among cultivars (Table 2). Comparative analysis indicated that the DAMs in THA vs. GTM, THA vs. YNH, and YNH vs. GTM included flavonoids that were notably enriched in flavonoid biosynthesis (ko00941), isoflavonoid biosynthesis (ko00943), and phenylpropanoid biosynthesis (ko00940) (Figure 4). These results are consistent with those of previous studies, which show that the pulp color of jackfruit is related to flavonoid biosynthesis (Cen et al. 2021). We further performed a quantitative analysis of the flavonoids in the pulp samples, identifying 48 flavonoids (Table 3). Comparative analysis identified 10 flavonoids predominantly accumulated in THA pulp, especially naringenin chalcone, eriodictyol, and taxifolin; 4 in GTM pulp, especially phlorizin; and 11 in YNH pulp, especially apigenin and luteolin, suggesting differences in the flavonoid composition of jackfruit pulps with various colors. Some of the flavonoids reported previously, including phloretin and hesperetin, displayed relatively low contents in this study.

In addition to flavonoids, lipid‐soluble carotenoids also contribute to the coloration of jackfruit pulp through varying components and contents (de Faria et al. 2009). Hu et al. 2017 demonstrated that *β*‐citraurin, luteoxanthin, and all‐trans‐neoxanthin were the main colorants in red‐flesh jackfruit, and violaxanthin, lutein, and 9‐cis‐violaxanthin were the main colorants in yellow‐flesh jackfruit. In the present study, we detected 46 carotenoids in the pulp of the three jackfruit cultivars (Table 4). Comparative analysis identified 15 carotenoids predominantly accumulated in THA pulp, especially lutein dimyristate, and 15 in YNH pulp, especially zeaxanthin, violaxanthin dipalmitate, violaxanthin‐myristate‐palmitate, and violaxanthin dimyristate. GTM pulp contained relatively low carotenoid content except for lutein, which also accumulated in THA pulp, suggesting its potential role in the yellow tone of pulp. The most abundant orange‐colored zeaxanthin (Tang et al. 2024) may have contributed greatly to the orange tone of YNH pulp. However, other carotenoids contributing to orange coloration (Fernández‐López et al. 2020), such as *α*‐carotene, *β*‐carotene, and *β*‐cryptoxanthin, showed a relatively low content in our study.

The inconsistency in metabolites content in jackfruit pulp could be attributed to differences in cultivars, environmental conditions, or analytical methods. Researches showed that, environmental factors such as shading, light, low temperature and hormone, could affect carotenoid biosynthesis (Zhao et al. 2022; Mao et al. 2025). Even though planted in the same germplasm nursery, different varieties and lines may exhibit variations in metabolic pathway activity. The red‐fleshed R‐M5, R‐Zhenzhuguo and R‐Sijihong showed differences in the content of flavonoids and carotenoids (Hu et al. 2017; Cen et al. 2021). Furthermore, advancements in metabolomic technologies and the expanding variety of available reference standards have made it more advantageous than ever to detect and quantify metabolites in jackfruit pulp.

Transcriptomic analysis identified 8078 DEGs between THA and GTM, 6754 DEGs between THA and YNH, and 7903 DEGs between YNH and GTM (Figure 9). In flavonoid biosynthesis, phenylalanine serves as a precursor to synthesize flavonoids. In GTM pulp, genes encoding *PAL* (*AHE.Chr26.1172*), *C4H* (*AHE.Chr19.24*), and *4CL* (*AHE.Chr20.345*), which catalyze the conversion of phenylalanine to *p*‐coumaroyl‐CoA, were highly expressed. Subsequently, *p*‐coumaroyl‐CoA is further converted into naringenin chalcone, naringenin, phlorizin, and caffeoyl‐CoA. The gene encoding *4CL* (*AHE.Chr28.135*) showed elevated expression in THA pulp. Genes encoding *CHI* (*AHE.Chr27.485*) and *HCT* (*AHE.Chr23.11*, *AHE.Chr23.12*) were highly expressed in YNH pulp, and those encoding *HCT* (*AHE.Chr13.1292*, *AHE.Chr04.1699*) were expressed in GTM pulp. The elevated expression of these genes in GTM and YNH likely redirected metabolic flux toward downstream products, thereby reducing the accumulation of naringenin chalcones and naringenin in their pulps. The subsequent reaction, catalyzed by F3H, produces dihydrokaempferol, which can be converted to taxifolin under the action of F3′5′H or to astragalin via FLS. The gene encoding *FLS* (*AHE.Chr05.909*) showed the highest expression in THA pulp. Finally, taxifolin is transformed into cyanidin under the consecutive action of DFR and ANS (Shen et al. 2022). Genes encoding *DFR* (*AHE.Chr*05.1351) and *ANS* (*AHE.Chr06.597*) showed elevated expression in THA and GTM pulps, respectively (Figure 10). Collectively, these results showed that flavonoid biosynthetic genes were expressed at lower levels in THA pulp than in GTM and YNH, except for those encoding *FLS*, *PAL*, and *4CL*. Furthermore, genes encoding *S3H* (*AHE.Chr09.1460* and *AHE.Chr09.1461*), which reduce the stimulatory effect of salicylic acid on flavonoid biosynthesis (Zhang et al. 2013; Yamamoto et al. 2020; Liu et al. 2023), were strongly expressed in THA pulp. This may explain the low expression levels of genes related to flavonoid biosynthesis in the THA pulp. In carotenoid biosynthesis, geranylgeranyl pyrophosphate serves as a precursor of carotenoids (Yu et al. 2024), and its conversion to phytoene, a colorless carotenoid, is catalyzed by PSY. The gene encoding *PSY* (*AHE.Chr22.1872*) was predominantly elevated in THA pulp. Phytoene was consecutively converted to lycopene under the action of PDS, Z‐ISO, ZDS, and CRT‐ISO. Genes encoding *PDS* (*AHE.Chr15.1439*) and *ZDS* (*AHE.Chr12.485*) accumulated in the THA pulp. The combined catalyzation of LCYE and LCYB, or the independent catalyzation of LCYB, transforms lycopene to *α*‐carotene and *β*‐carotene respectively. The gene encoding *LCYE* was highly expressed in THA and YNH pulp during this process. *α*‐carotene is then converted to lutein under the catalysis of LUT1 and LUT5, and *β*‐carotene is converted to *β*‐cryptoxanthin and zeaxanthin under the actions of BCH and LUT5. The gene encoding *LUT5* (*AHE.Chr11.451*) accumulated in the THA pulp, thus producing a relatively higher lutein content. Subsequently, ZEP catalyzes the synthesis of violaxanthin from zeaxanthin, whereas VDE can de‐epoxidate violaxanthin into epoxide‐free zeaxanthin (Leonelli 2022). During this process, the gene expression of *ZEP* (*AHE.Chr01.998*, *AHE.Chr02.742*) and *VDE* (*AHE.Chr01.273*) was active in the THA pulp. In GTM pulp, the highly expressed *ZEP* (*AHE.Chr16.1073*) played a major role in this catalytic reaction. Finally, NSY catalyzes the conversion of violaxanthin to neoxanthin, which is further degraded into ABA precursors (Figure 11). The results showed that, in contrast to the biosynthetic pattern of flavonoids, the expression of genes related to carotenoid biosynthesis in THA pulp was generally active.

## 5 Conclusions

In conclusion, this study employed a combined transcriptomic and metabolomic analysis to elucidate the molecular basis of pulp coloration in jackfruit. We identified several DEGs associated with pigmentation, among which *PAL*, *C4H*, *4CL*, *F3H*, *FLS*, *ANS*, and *ANR* played major roles in flavonoid biosynthesis, while *PSY*, *PDS*, *ZDS*, *LCYE*, *LUT5*, *ZEP*, and *VDE* were key contributors to carotenoid biosynthesis. Metabolomic profiling further identified the specific flavonoids and carotenoids responsible for pulp color: naringenin chalcone, eriodictyol, taxifolin, zeaxanthin, and lutein were enriched in the light‐yellow THA pulp; phlorizin and lutein were predominant in the yellow tone of GTM pulp; and apigenin, luteolin, zeaxanthin, and violaxanthin dipalmitate contributed to the orange tone of YNH pulp. Together, these results highlight the coordinated roles of flavonoids, carotenoids, and their regulatory genes in shaping the diverse coloration patterns of jackfruit pulp. The findings provide valuable insights and offer new prospects for the genetic improvement of the quality of jackfruit.

## Author Contributions

**Jianjun Liang:** conceptualization, methodology, writing – original draft, investigation, data curation. **Xiangwei Ma:** methodology, formal analysis. **Chenxin Yi:** investigation, resources. **Hailan Zhou:** investigation, software. **Zhuangmin Wei:** visualization, resources. **Xiuguan Tang:** visualization, investigation. **Weiyan Ye:** investigation, resources. **Hailing Tang:** visualization, resources. **Pengjin Zhu:** writing – review and editing, project administration, investigation, funding acquisition.

## Funding

This research was funded by the Special Project for Basic Scientific Research Business of Guangxi Academy of Agricultural Sciences (GNK 2025YP128 and GNK 2026YT021), and the vanguard team of the specialty fruit industry GNKM (202504‐03), and Guangxi Key Research and Development Program (GK AB21220003).

## Ethics Statement

The authors have nothing to report.

## Conflicts of Interest

The authors declare no conflicts of interest.

## Supporting information


**Table S1:** List of flavonoids detected in this research.
**Table S2:** List of carotenoids detected in this research.
**Table S3:** Transcriptomic data filtering.
**Table S4:** List of differentially expressed genes in the comparison groups.
**Table S5:** The co‐expression analysis of DEGs and DAFs or DACs based on Pearson correlation.
**Figure S1:** Pearson's correlation coefficient for metabolome data (a) and transcriptome data (b).
**Figure S2:** KEGG enrichment analysis of DEGs in THA vs. GTM.
**Figure S3:** GO enrichment analysis of DEGs in THA vs. GTM.
**Figure S4:** KEGG enrichment analysis of DEGs in THA vs. YNH.
**Figure S5:** GO enrichment analysis of DEGs in THA vs. YNH.
**Figure S6:** KEGG enrichment analysis of DEGs in YNH vs. GTM.
**Figure S7:** GO enrichment analysis of DEGs in YNH vs. GTM.

## Data Availability

The datasets generated and analyzed for this study can be found in the global pharmacopeia genome database (http://www.gpgenome.com/species/92). Raw reads for RNA‐Seq were downloaded from the NCBI database with accession number SRP129502 and SRP092562.
