# Supplementary figures and images for: Combined Analysis of Transcriptome and Metabolome Explains the Differences in Pulp Color in Jackfruit
Source: Plant Direct. 2026 Feb 11;10(2):e70127. doi: 10.1002/pld3.70127 (PMC12894421; doi:10.1002/pld3.70127)

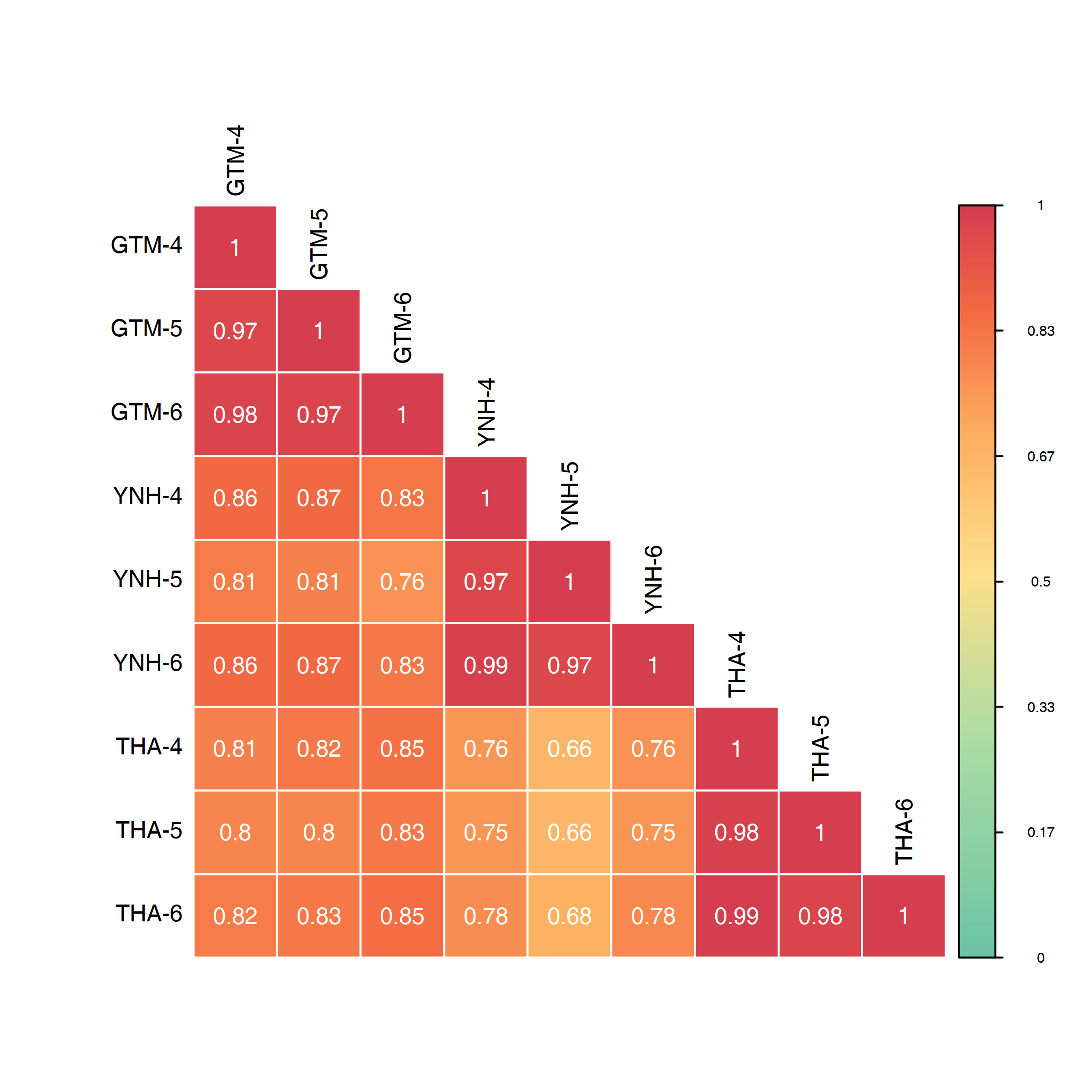

Supplement: Supplementary file 1 — Table S1: List of flavonoids detected in this research. Table S2: List of carotenoids detected in this research. Table S3: Transcriptomic data filtering. Table S4: List of differentially expressed genes in the comparison groups. Table S5: The co‐expression analysis of DEGs and DAFs or DACs based on Pearson correlation. Figure S1: Pearson's correlation coefficient for metabolome data (a) and transcriptome data (b). Figure S2: KEGG enrichment analysis of DEGs in THA vs. GTM. Figure S3: GO enrichment analysis of DEGs in THA vs. GTM. Figure S4: KEGG enrichment analysis of DEGs in THA vs. YNH. Figure S5: GO enrichment analysis of DEGs in THA vs. YNH. Figure S6: KEGG enrichment analysis of DEGs in YNH vs. GTM. Figure S7: GO enrichment analysis of DEGs in YNH vs. GTM. [file PLD3-10-e70127-s001.zip › Supplementary Figure S1a.png]

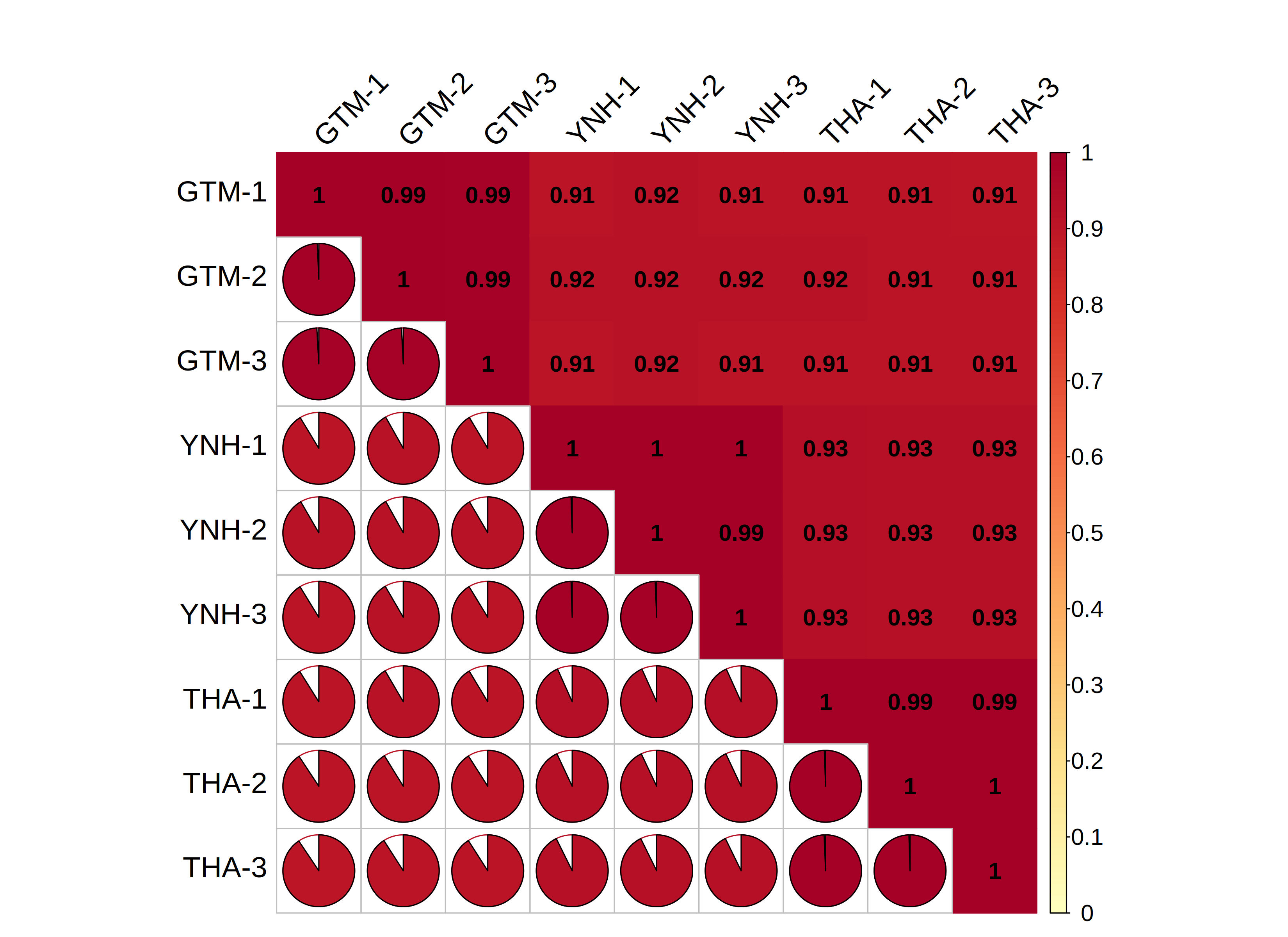

Supplement: Supplementary file 1 — Table S1: List of flavonoids detected in this research. Table S2: List of carotenoids detected in this research. Table S3: Transcriptomic data filtering. Table S4: List of differentially expressed genes in the comparison groups. Table S5: The co‐expression analysis of DEGs and DAFs or DACs based on Pearson correlation. Figure S1: Pearson's correlation coefficient for metabolome data (a) and transcriptome data (b). Figure S2: KEGG enrichment analysis of DEGs in THA vs. GTM. Figure S3: GO enrichment analysis of DEGs in THA vs. GTM. Figure S4: KEGG enrichment analysis of DEGs in THA vs. YNH. Figure S5: GO enrichment analysis of DEGs in THA vs. YNH. Figure S6: KEGG enrichment analysis of DEGs in YNH vs. GTM. Figure S7: GO enrichment analysis of DEGs in YNH vs. GTM. [file PLD3-10-e70127-s001.zip › Supplementary Figure S1b.tiff]

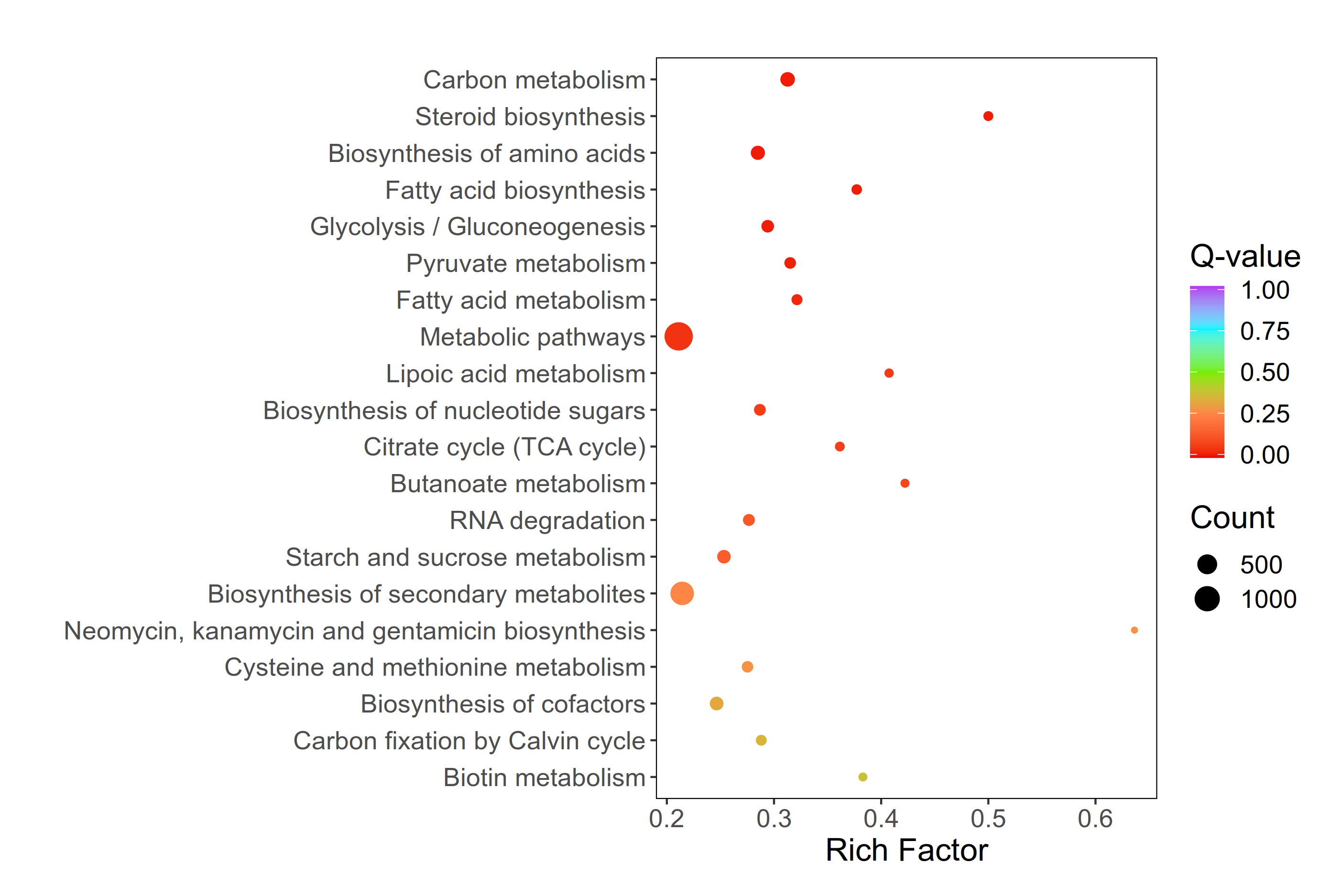

Supplement: Supplementary file 1 — Table S1: List of flavonoids detected in this research. Table S2: List of carotenoids detected in this research. Table S3: Transcriptomic data filtering. Table S4: List of differentially expressed genes in the comparison groups. Table S5: The co‐expression analysis of DEGs and DAFs or DACs based on Pearson correlation. Figure S1: Pearson's correlation coefficient for metabolome data (a) and transcriptome data (b). Figure S2: KEGG enrichment analysis of DEGs in THA vs. GTM. Figure S3: GO enrichment analysis of DEGs in THA vs. GTM. Figure S4: KEGG enrichment analysis of DEGs in THA vs. YNH. Figure S5: GO enrichment analysis of DEGs in THA vs. YNH. Figure S6: KEGG enrichment analysis of DEGs in YNH vs. GTM. Figure S7: GO enrichment analysis of DEGs in YNH vs. GTM. [file PLD3-10-e70127-s001.zip › Supplementary Figure S2 .tiff]

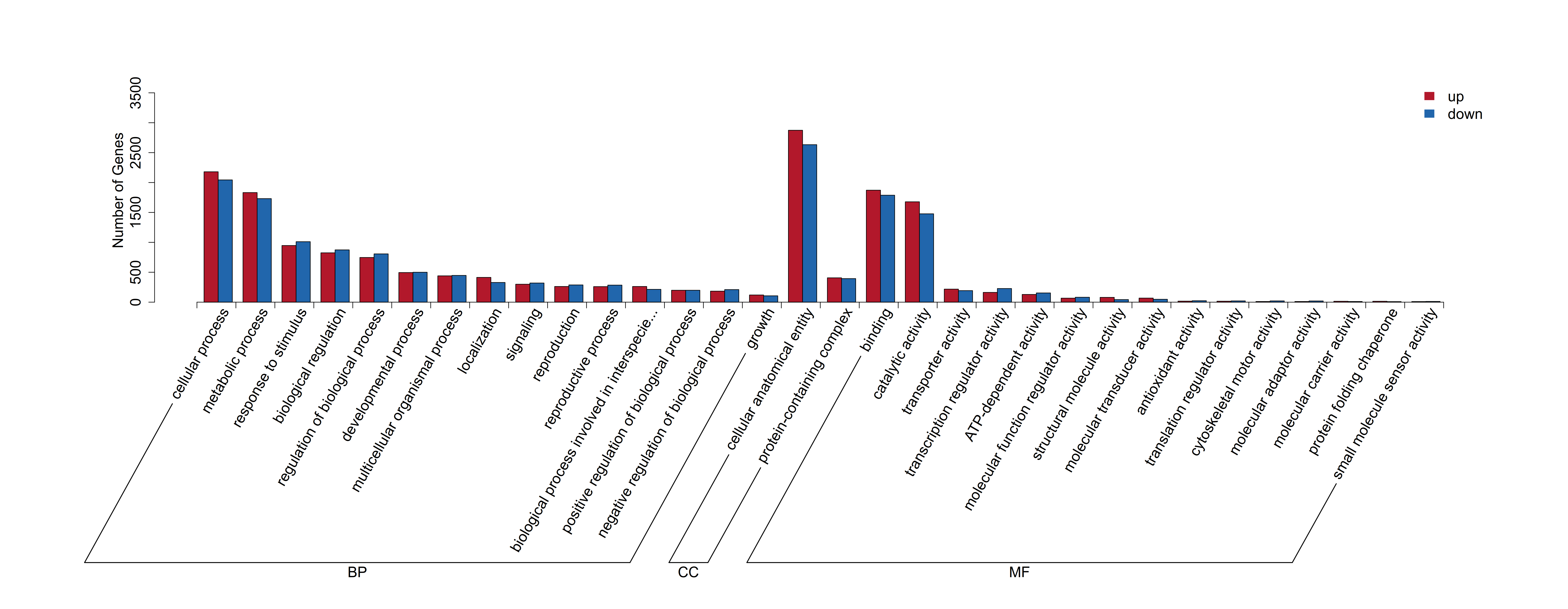

Supplement: Supplementary file 1 — Table S1: List of flavonoids detected in this research. Table S2: List of carotenoids detected in this research. Table S3: Transcriptomic data filtering. Table S4: List of differentially expressed genes in the comparison groups. Table S5: The co‐expression analysis of DEGs and DAFs or DACs based on Pearson correlation. Figure S1: Pearson's correlation coefficient for metabolome data (a) and transcriptome data (b). Figure S2: KEGG enrichment analysis of DEGs in THA vs. GTM. Figure S3: GO enrichment analysis of DEGs in THA vs. GTM. Figure S4: KEGG enrichment analysis of DEGs in THA vs. YNH. Figure S5: GO enrichment analysis of DEGs in THA vs. YNH. Figure S6: KEGG enrichment analysis of DEGs in YNH vs. GTM. Figure S7: GO enrichment analysis of DEGs in YNH vs. GTM. [file PLD3-10-e70127-s001.zip › Supplementary Figure S3.tiff]

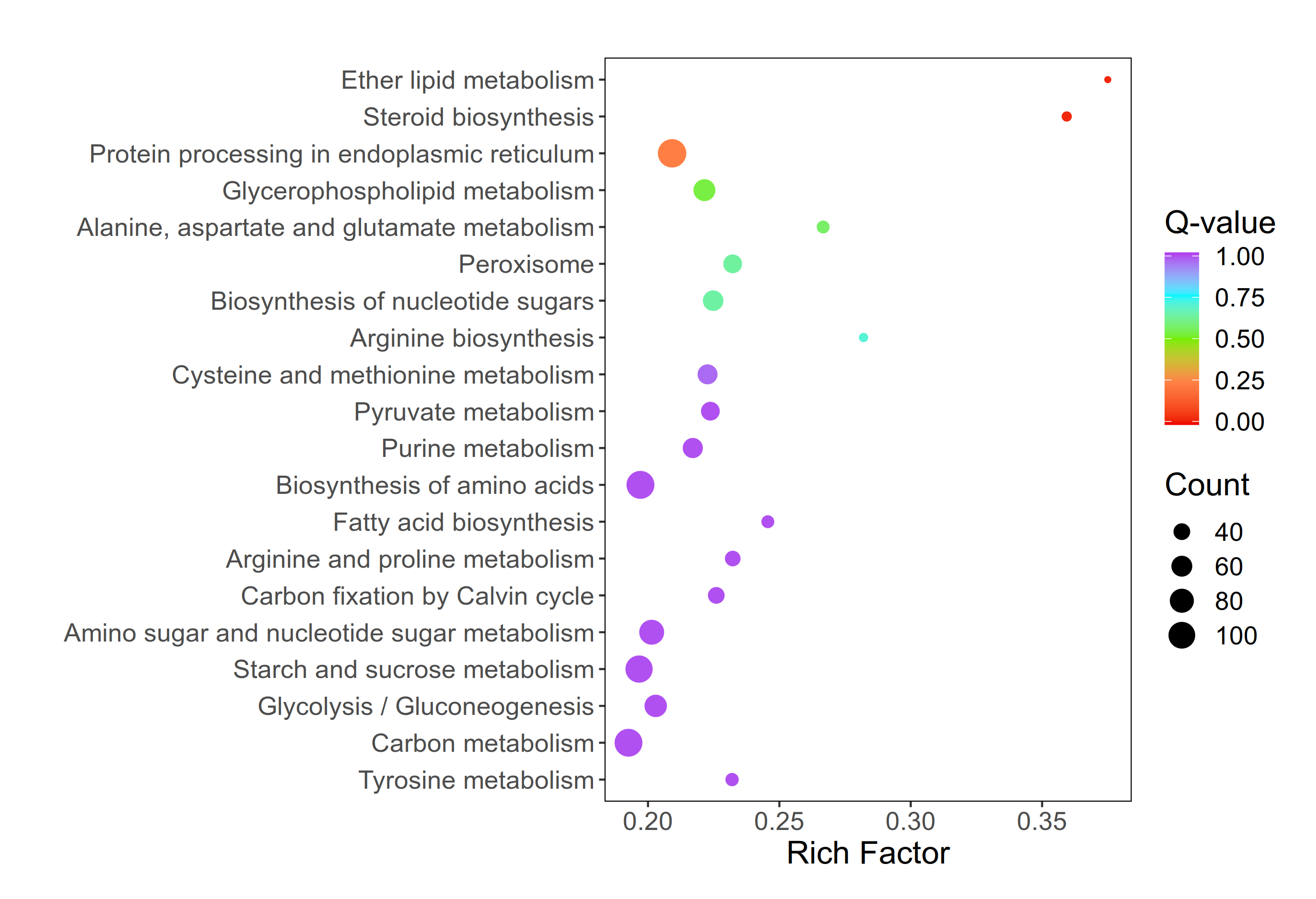

Supplement: Supplementary file 1 — Table S1: List of flavonoids detected in this research. Table S2: List of carotenoids detected in this research. Table S3: Transcriptomic data filtering. Table S4: List of differentially expressed genes in the comparison groups. Table S5: The co‐expression analysis of DEGs and DAFs or DACs based on Pearson correlation. Figure S1: Pearson's correlation coefficient for metabolome data (a) and transcriptome data (b). Figure S2: KEGG enrichment analysis of DEGs in THA vs. GTM. Figure S3: GO enrichment analysis of DEGs in THA vs. GTM. Figure S4: KEGG enrichment analysis of DEGs in THA vs. YNH. Figure S5: GO enrichment analysis of DEGs in THA vs. YNH. Figure S6: KEGG enrichment analysis of DEGs in YNH vs. GTM. Figure S7: GO enrichment analysis of DEGs in YNH vs. GTM. [file PLD3-10-e70127-s001.zip › Supplementary Figure S4.tiff]

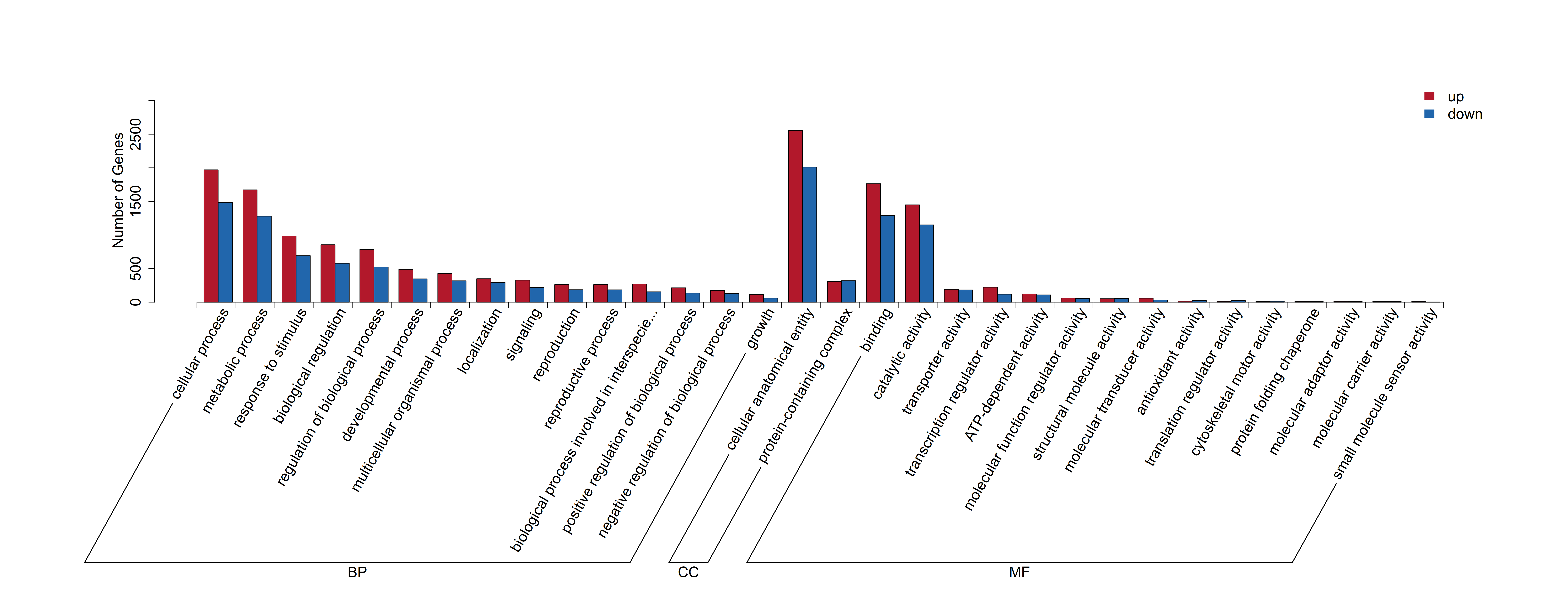

Supplement: Supplementary file 1 — Table S1: List of flavonoids detected in this research. Table S2: List of carotenoids detected in this research. Table S3: Transcriptomic data filtering. Table S4: List of differentially expressed genes in the comparison groups. Table S5: The co‐expression analysis of DEGs and DAFs or DACs based on Pearson correlation. Figure S1: Pearson's correlation coefficient for metabolome data (a) and transcriptome data (b). Figure S2: KEGG enrichment analysis of DEGs in THA vs. GTM. Figure S3: GO enrichment analysis of DEGs in THA vs. GTM. Figure S4: KEGG enrichment analysis of DEGs in THA vs. YNH. Figure S5: GO enrichment analysis of DEGs in THA vs. YNH. Figure S6: KEGG enrichment analysis of DEGs in YNH vs. GTM. Figure S7: GO enrichment analysis of DEGs in YNH vs. GTM. [file PLD3-10-e70127-s001.zip › Supplementary Figure S5.tiff]

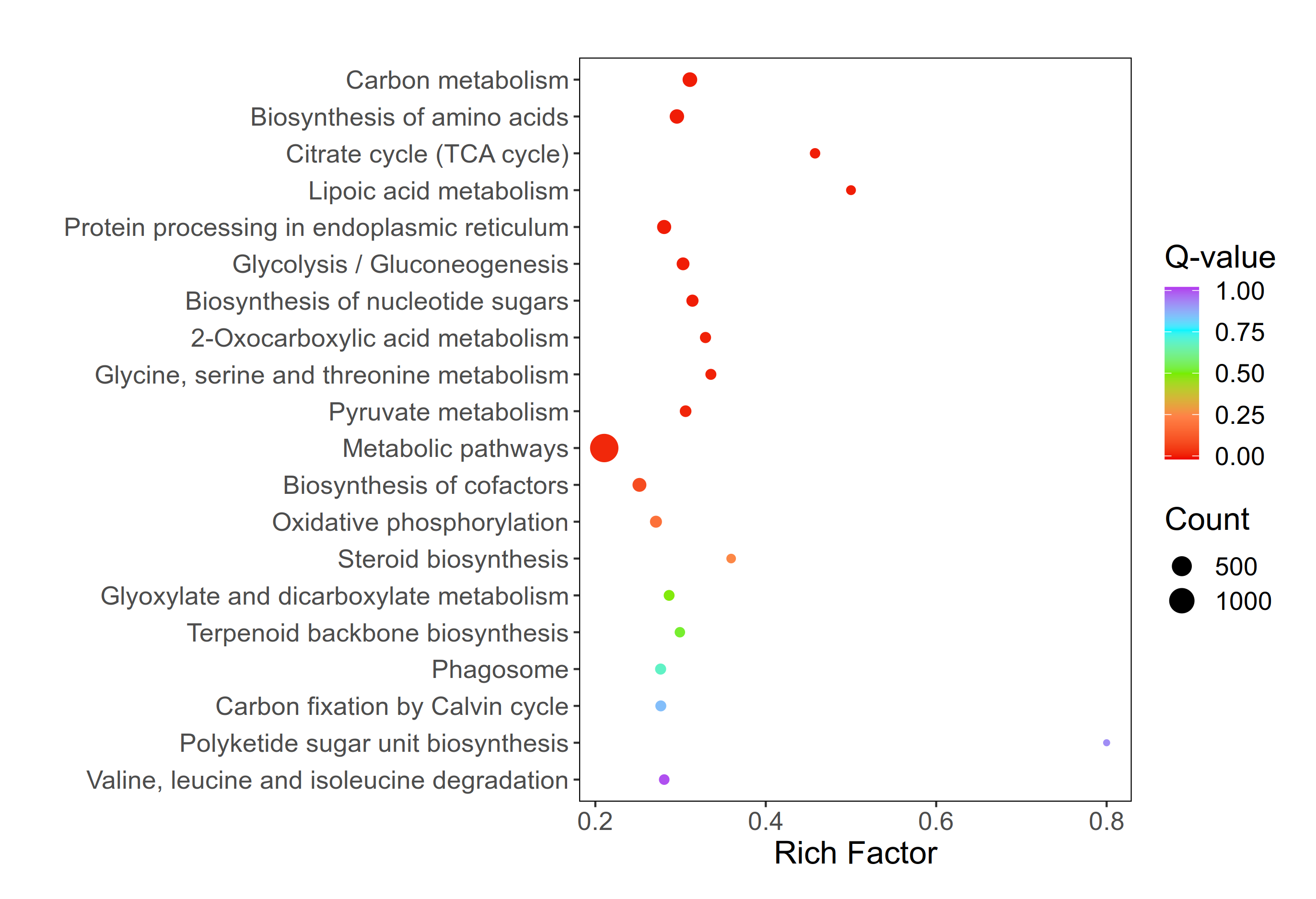

Supplement: Supplementary file 1 — Table S1: List of flavonoids detected in this research. Table S2: List of carotenoids detected in this research. Table S3: Transcriptomic data filtering. Table S4: List of differentially expressed genes in the comparison groups. Table S5: The co‐expression analysis of DEGs and DAFs or DACs based on Pearson correlation. Figure S1: Pearson's correlation coefficient for metabolome data (a) and transcriptome data (b). Figure S2: KEGG enrichment analysis of DEGs in THA vs. GTM. Figure S3: GO enrichment analysis of DEGs in THA vs. GTM. Figure S4: KEGG enrichment analysis of DEGs in THA vs. YNH. Figure S5: GO enrichment analysis of DEGs in THA vs. YNH. Figure S6: KEGG enrichment analysis of DEGs in YNH vs. GTM. Figure S7: GO enrichment analysis of DEGs in YNH vs. GTM. [file PLD3-10-e70127-s001.zip › Supplementary Figure S6.tiff]

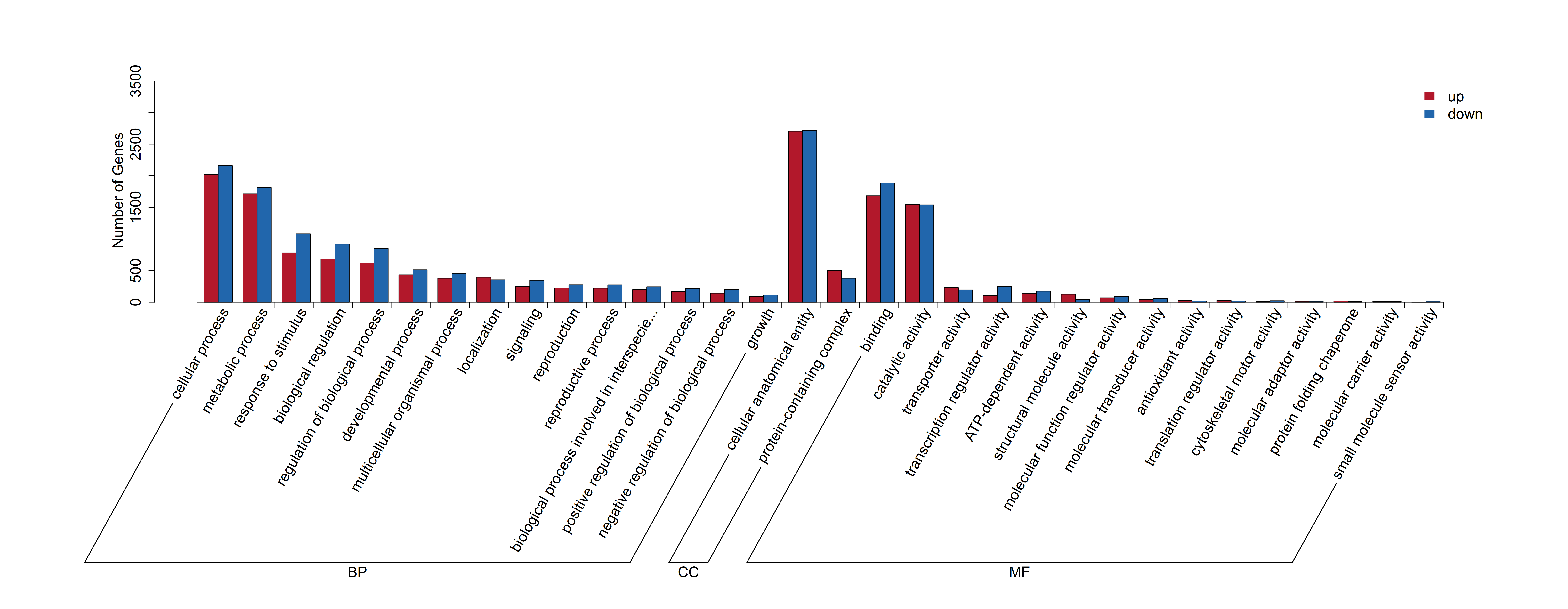

Supplement: Supplementary file 1 — Table S1: List of flavonoids detected in this research. Table S2: List of carotenoids detected in this research. Table S3: Transcriptomic data filtering. Table S4: List of differentially expressed genes in the comparison groups. Table S5: The co‐expression analysis of DEGs and DAFs or DACs based on Pearson correlation. Figure S1: Pearson's correlation coefficient for metabolome data (a) and transcriptome data (b). Figure S2: KEGG enrichment analysis of DEGs in THA vs. GTM. Figure S3: GO enrichment analysis of DEGs in THA vs. GTM. Figure S4: KEGG enrichment analysis of DEGs in THA vs. YNH. Figure S5: GO enrichment analysis of DEGs in THA vs. YNH. Figure S6: KEGG enrichment analysis of DEGs in YNH vs. GTM. Figure S7: GO enrichment analysis of DEGs in YNH vs. GTM. [file PLD3-10-e70127-s001.zip › Supplementary Figure S7.tiff]
